# A comprehensive monitoring and evaluation framework for evidence to policy networks

**DOI:** 10.1016/j.evalprogplan.2022.102053

**Published:** 2022-02-18

**Authors:** Tanja Kuchenmüller, Evelina Chapman, Ryoko Takahashi, Louise Lester, Marge Reinap, Moriah Ellen, Michelle M. Haby

**Affiliations:** a WHO Regional Office for Europe, Copenhagen, Denmark; b Public Health, Nottinghamshire County Council, West Bridgford, Nottinghamshire, United Kingdom; c Department of Health Policy and Management, Guilford Glazer Faculty of Business and Management and Faculty of Health Sciences, Ben-Gurion University of the Negev, Israel; d Departamento de Ciencias Químico Biológicas, Universidad de Sonora, Hermosillo, Sonora, Mexico; e Centre for Health Policy, Melbourne School of Population and Global Health, The University of Melbourne, Melbourne, Victoria, Australia

## Abstract

**Objective:**

To describe the development of a framework for monitoring and evaluating knowledge translation (KT) networks.

**Method:**

The framework was developed using mixed methods over four phases, including i) a targeted literature review of KT networks, activities and indicators, ii) two scoping reviews to further enhance the set of indicators, iii) peer-reviews by international KT experts and an online expert consultation, and iv) piloting.

**Results:**

A comprehensive theory of change (ToC) and indicators, both for the Network Secretariat and its participating member countries, were identified to develop the monitoring and evaluation framework. The framework includes (i) a ToC, including three key indicator domains across the results chain (outputs, short term outcomes, intermediate outcomes), and (ii) indicators for the three key domains, that can be selected depending on the stage of network maturity, along with suggested data collection methods. The three key indicator domains are 1) KT capacity and skill building; 2) network (structure, governance and leadership); and 3) KT/evidence-informed policy value and culture.

**Conclusion:**

The monitoring and evaluation framework that *links KT activities with policy and health outcomes* fills an important gap in optimizing KT procedures, generating lessons learned and increasing accountability of major multipartner KT networks.

## 1 Introduction

The use of sound evidence to inform public health policy is likely to ensure greater and more equitable population health gains (Shaxson, Datta, Tshangela, & Matomela, 2016; van de Goor et al., 2017) and optimize resource allocation (Lavis et al., 2003). This has increased global interest in the role that knowledge translation (KT) can play in informing policy (Grimshaw, Eccles, Lavis, Hill, & Squires, 2012; WHO Regional Office for Europe, 2016a). In parallel, research into new models and mechanisms of KT has expanded (Oliver, Innvar, Lorenc, Woodman, & Thomas, 2014; Shaxson et al., 2016). KT is defined as: “the exchange, synthesis, and effective communication of reliable and relevant research results. The focus is on promoting interaction among the producers and users of research, removing the barriers to research use, and tailoring information to different target audiences so that effective interventions are used more widely”(World Health Organization, 2004).

Translating research into policy is recognized as a complex and iterative process (Lavis et al., 2012; Moat, Lavis, & Abelson, 2013; Woolf, 2008). Acknowledging the political nature of decision-making (Mair, 2019), a systems approach is required that shifts attention from assessing individual components (such as change in KT capacity at an individual level) to the dynamic interfaces of the components (including structures) in a complex system (Best et al., 2009).

Networks, such as the Evidence-informed Policy Network (EVIPNet) Europe, use a systems thinking approach (Willis, Riley, Best, & Ongolo-Zogo, 2012) and play a critical role in facilitating KT processes (Mendizabal, 2006; Perkin & Court, 2005). Other examples of KT networks include the global multi-year programme, Building Capacity to Use Research Evidence (BCURE). The BCURE programme developed organizational knowledge brokering projects in 12 low- and middle-income countries between 2013 and 2017 spanning beyond health. In addition, the Partnership for Evidence and Equity in Responsive Social Systems (PEERSS), funded by the International Development and Research Center (IDRC) and the William and Flora Hewlett Foundation, is aiming to advance evidence-informed policy-making (EIP) in the social systems. While these initiatives are multi-sectoral in nature, the Regional East African Community Health Policy Initiative (REACH-PI) (Campbell, 2013; East African Community, 2006) is a subregional KT network that bridges the gap between health research and policy and decision making through the establishment of Knowledge Translation Platforms (KTPs) in Kenya, Tanzania and Uganda. As a result of the similar mandate, REACH-PI and EVIPNet established a close interaction. The establishment of EVIPNet Africa was inspired by the preparatory work undertaken by REACH-PI, which then became a part of the EVIPNet Africa as a subregion (Lavis & Panisset, 2010). EVIPNet Africa also explicitly supported the REACH-PI’s Rapid Response Service in Uganda, which was then rolled out into other countries within the African region and in other regions (World Health Organization, 2016).

To date, few evaluations have been conducted of organizational or system-level KT activities (Dobbins, Traynor, Workentine, Yousefi-Nooraie, & Yost, 2018; Haby et al., 2016; Lester, Haby, Chapman, & Kuchenmüller, 2020; The CIPHER Investigators, 2014; Willis et al., 2012). In part, this may be due to the complexity and context specific nature of KT activities, which make them challenging to evaluate (The CIPHER Investigators, 2014; Van Eerd et al., 2011). In addition, the lack of evaluation infrastructure and knowledge, and challenges and concerns about how findings will be acted on (Lavis et al., 2003) may further explain the limited KT evaluation activity.

This paper describes the development of a comprehensive monitoring and evaluation (M&E) framework for EVIPNet Europe, which is meant to capture KT activities for EIP at both the Network Secretariat and the individual country team levels. While the framework was developed in the context of EVIPNet Europe, it is meant to serve also other KT networks or programmes at national, regional or global levels.

## 2 EVIPNet Europe

In 2005, as a response to Member States’ request, the World Health Organization (WHO) launched EVIPNet as a KT capacity building network (Hamid et al., 2005). It has the objective of improving public health and reducing inequities by supporting Member States in routinely using the best available scientific evidence in policy development (WHO Regional Office for Europe, 2015).

Since 2005, regional EVIPNet networks have been created in Asia, the Americas, sub-Saharan Africa, and the Eastern Mediterranean to support Member States in this endeavour. In 2012, the WHO European Region launched EVIPNet Europe, which is the most recent EVIPNet regional network (WHO Regional Office for Europe, 2015), to accelerate the achievement of important health goals such as the Millennium Development Goals (World Health Organization, 2005) and the subsequent Sustainable Development Goals (World Health Organization/General Assembly, 2015). As of 2020, 23 countries were part of EVIPNet Europe. Additional details on the strategic directions and approaches of EVIPNet Europe can be found in the EVIPNet Europe Strategic Plan (WHO Regional Office for Europe, 2015), which has served as the roadmap for the implementation of the network.

EVIPNet Europe aims to address the substantial gap between what is scientifically known and what is done (Straus, Tetroe, & Graham, 2009). The ultimate goal, as expressed in the network’s vision, is to ensure that high-quality, context-sensitive evidence is routinely used to inform health decision-making processes to strengthen health outcomes across the Region (WHO Regional Office for Europe, 2015). Key to achieving this goal is to bring together national health policy-makers, managers, researchers, members of civil society, practitioners who operate on multiple levels to advance and facilitate policy development and implementation informed by the best available evidence (WHO Regional Office for Europe, 2015). These country teams – so-called KTPs (El-Jardali, Bou-Karroum, & Fadlallah, 2020)^1^ – plan, implement, monitor and evaluate KT interventions at country level, including priority-setting exercises, evidence briefs for policy (EBPs), policy dialogue exercises (PDs), rapid response services, clearinghouses and capacity-building (WHO Regional Office for Europe, 2017).

The changes initiated by EVIPNet in view of strengthening EIP are based on the strategic directions and cross-cutting approaches defined in the EVIPNet Europe Strategic Plan. They cover the following three domains (WHO Regional Office for Europe, 2015):

1) KT capacity and skill building: responding to the need to strengthen the KT capacity throughout the WHO European Region, EVIPNet Europe provides technical assistance, mentorships and exchanges, plus routine capacity-building workshops to improve the skill base of its network members.
2) Network structure, governance and leadership: regional network and governance structures have been established at regional level to provide leadership and facilitate network functioning. In addition, EVIPNet Europe assists in the establishment of country teams/KTPs to drive country-level activities. Country teams are national networks dedicated to strengthening innovative health partnerships among researchers, policy-makers and civil society.
3) KT and EIP value and culture: recognizing that country teams/KTPs will be most successful and sustainable in an enabling environment, EVIPNet Europe aims to increase awareness and commitment to improve the culture and practice of KT and EIP.

**Fig. 1:**
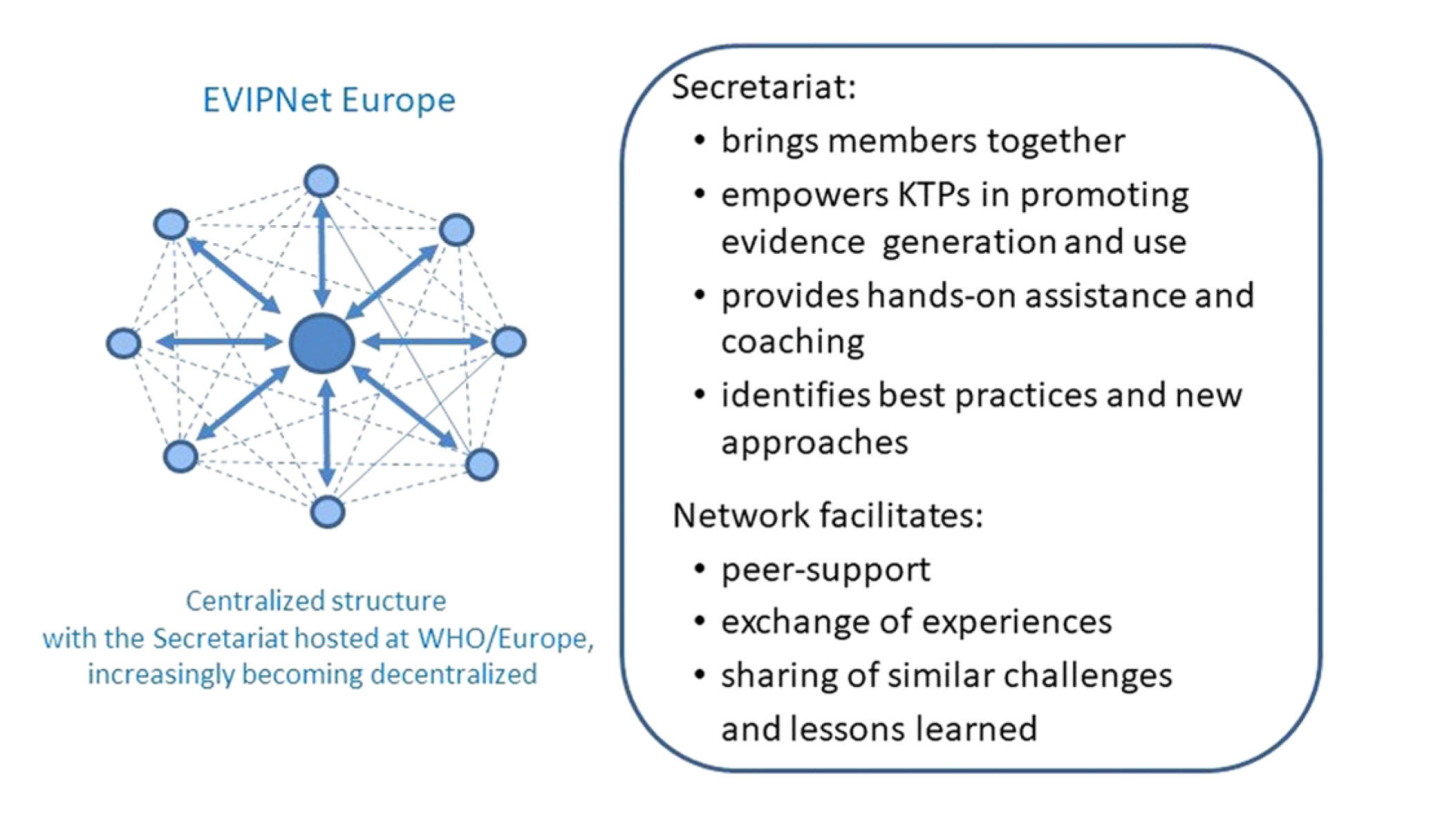
EVIPNet Europe – network structure. Source: Adapted from Starkley 1997 in Ramalingam (2011).

In the early stage of the network, EVIPNet Europe functioned as a centralized network, i.e. the WHO Secretariat is a hub connecting and coordinating the country teams/KTPs – serving as network nodes. The network builds vertical and horizontal linkages, which strengthen and amplify the culture and practice of KT (see Fig. 1). With time, it is expected that the network will gradually become more decentralized, with the nodes of the network increasingly interconnected and mutually supportive, reducing the central facilitation role of the WHO Secretariat.

**Fig. 2:**
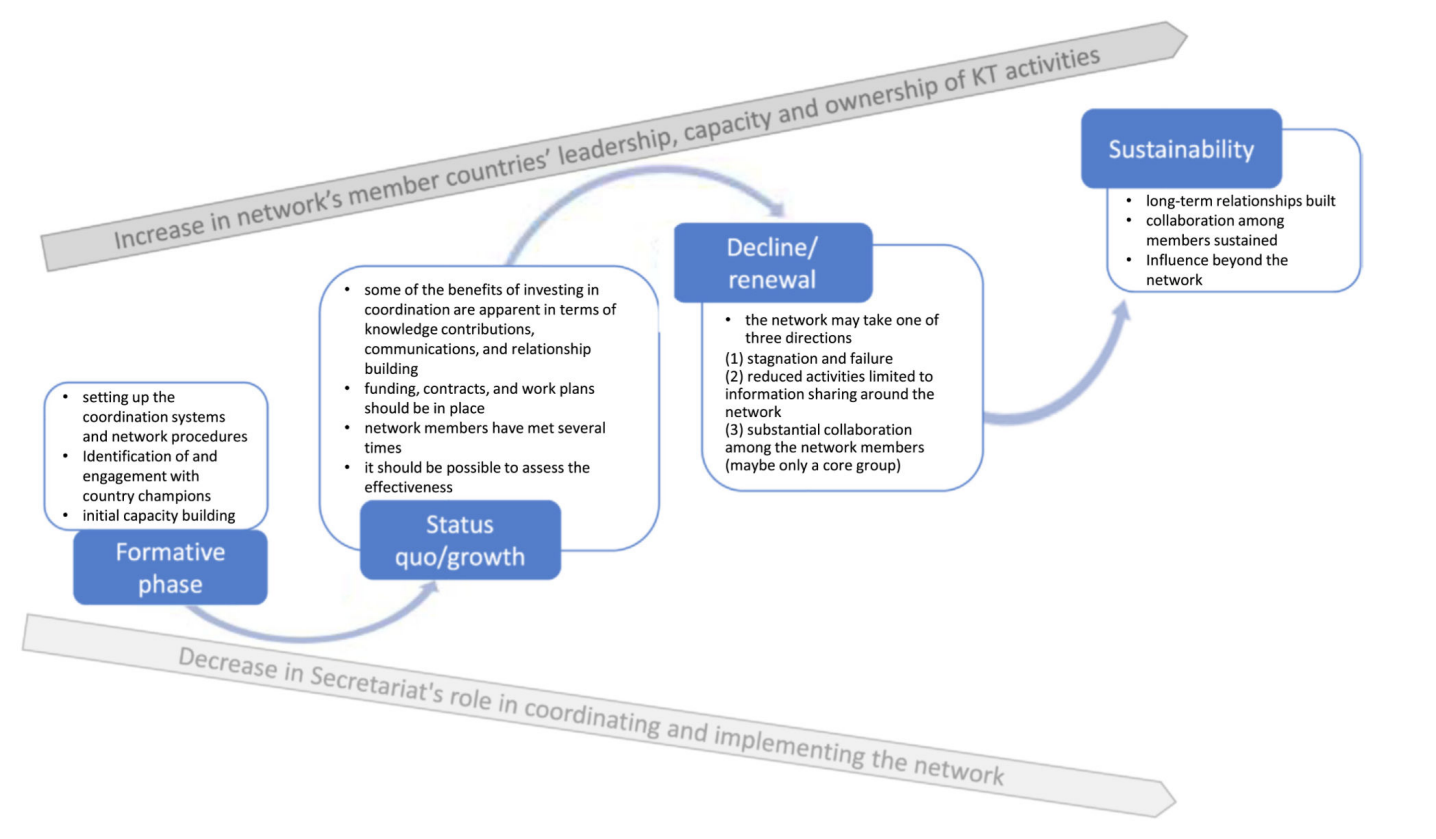
Network life cycle. Source: Based on Creech and Ramji (2004).

Like similar networks, the EVIPNet Europe network life-cycle (see Fig. 2) is expected to include a formative phase; followed by a second phase of status quo and/or growth; a third phase of stagnation, decline or renewal as the role of the Secretariat decreases; and finally, a sustainability phase in line with the increased role of member countries, during which continuity and increased country ownership is ensured (Creech & Ramji, 2004).

During the initiation and initial growth phase of the network (Fig. 2), the WHO Secretariat plays a strong and leading role. The WHO Secretariat regularly convenes the country teams/KTPs (virtually or in person), coordinates the network activities and offers technical support to individual countries with the aim of strengthening and institutionalizing knowledge brokering (see textbox in Fig. 1). Network member countries inter-connect horizontally: from early on, in the establishment of the Network, peer-support and mentoring as well as the exchange of experience between network members has been promoted. Throughout network maturation (Fig. 2), the leadership and coordination role of the network is expected to be increasingly transferred from the Secretariat to the network and country teams/KTPs. This encourages the KT process to be locally owned and, thus, more sustainable (Bennett & Jessani, 2011).

EVIPNet Europe embraces M&E and has subscribed to the principle of being a learning organization that seeks to regularly assess progress, identify good practices and lessons learned in order to improve and optimize performance (WHO Regional Office for Europe, 2015). Overall, WHO defines monitoring as “the routine tracking and reporting of priority information about a programme and its intended outputs and outcomes” (World Health Organization, 2011, p. V) and evaluation as the “rigorous, science-based analysis of information about programme activities, characteristics, outcomes and impact that determines the merit or worth of a specific programme or intervention” (World Health Organization, 2011, p. V).

In line with its focus on M&E, EVIPNet Europe also encourages critical evaluative thinking (WHO Regional Office for Europe, 2016b) and sharing of lessons learnt among network members (Scarlett et al., 2018; WHO Regional Office for Europe, 2019). Through its M&E work, EVIPNet Europe aims to (WHO Regional Office for Europe, 2015):

- ensure transparency and accountability of its activities;
- measure progress, effectiveness and efficiency of its strategy and activity implementation;
- facilitate the identification of implementation challenges and improve operations;
- enable network-wide knowledge sharing and learning; and
- contribute to KT research by creating evidence about which strategies are effective to inform future work and scaling-up.

## 3 Methods

**Fig. 3:**
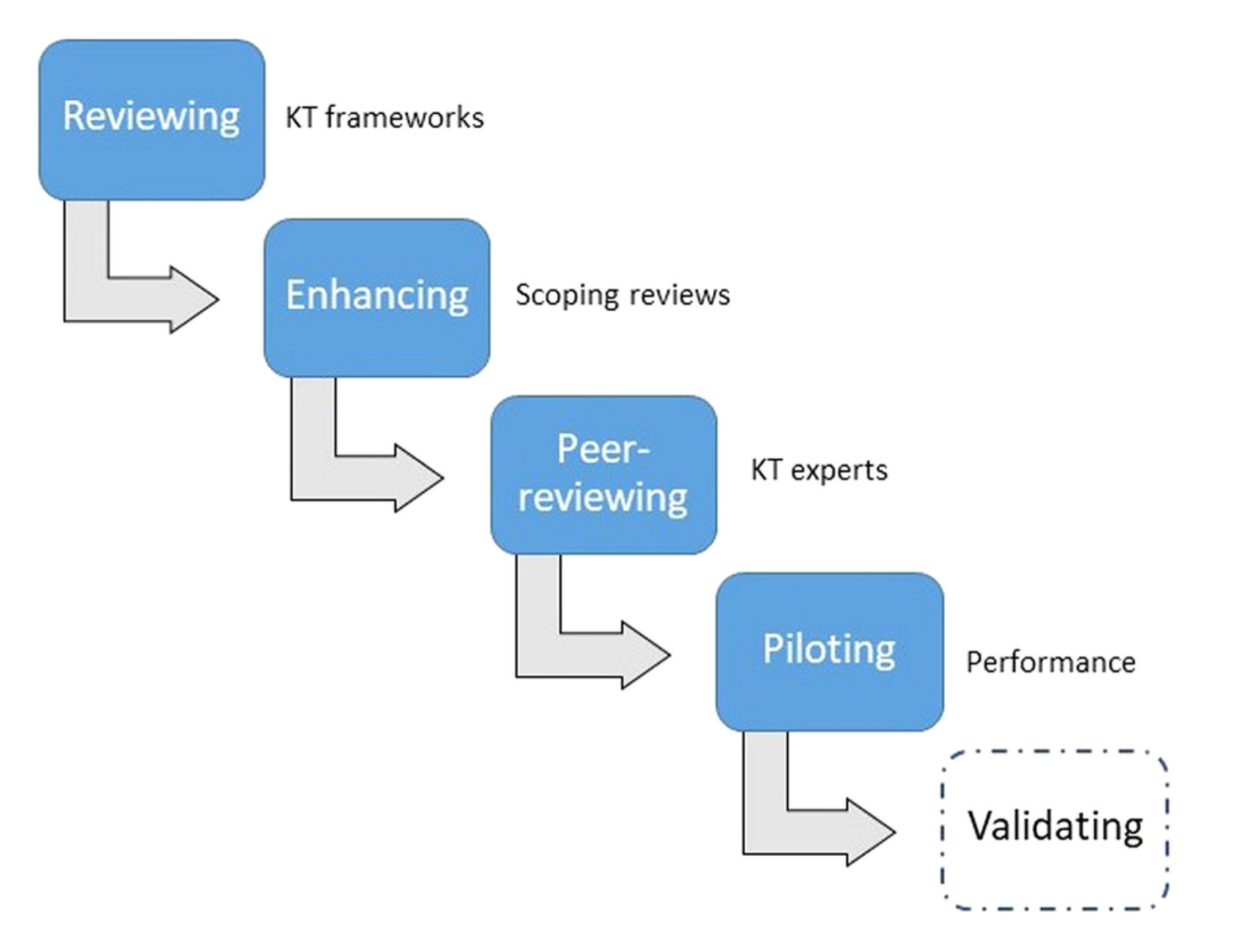
Development process for the EVIPNet Europe M&E framework. The user validation of the framework will be addressed in the next stage of the M&E framework development.

Mixed methods were used for the development of the EVIPNet Europe M&E framework. An overview of the process is shown in Fig. 3.

First, a targeted literature review was conducted of the M&E of knowledge (translation) networks and KT activities. This was combined with the experience and expert knowledge of the study team and used to develop a draft conceptual framework. The Framework includes: (i) a theory of change (ToC), including three key indicator domains across the results chain (outputs, short term outcomes, intermediate outcomes) and (ii) measures (indicators) for the three key domains, along with suggested data collection methods.

Outcome mapping (Earl, Carden, & Smutylo, 2001) served as an underlying approach to the framework development. A key principle of the outcome mapping methodology is its focus on contribution rather than attribution. In other words, instead of claiming to achieve impact, outcome mapping focuses on a programme’s influence on outcomes. These outcomes include changes in behaviours, actions and relationships. A programme’s outcomes can, subsequently and in the longer term, then heighten the possibility of achieving impacts. However, this relationship is not necessarily one of direct cause and effect due to the complexity of change processes and the involvement of multiple actors. The construction of the results chain in the ToC followed the logical framework (log frame) approach (Morton, Shaxson, & Greenland, 2012), which describes a succession of elements and causal interlinkages between them (Jones, 2011). It includes the assumptions, inputs, outputs and short- and intermediate-term outcomes.

This initial draft framework was submitted for peer-review by international KT and M&E experts to assess the framework’ s relevance and applicability. The initial framework suggested 72 Secretariat indicators and 73 country team/KTP indicators in line with the proposed ToC. For this, and subsequent revisions, the indicators were purposively selected based on relevance for measuring the success of EVIPNet Europe’s activities, and alignment with the ToC.

For the second phase of the process, two scoping reviews were undertaken to inform the development of a comprehensive set of evidence-based indicators and further enhance the framework. One was on country-level/KTP indicators (the detailed methodology is published elsewhere, see (Scarlett, Forsberg, Biermann, Kuchenmüller, & El-Khatib, 2020)) and the other on indicators related to the work of the Network Secretariat (see Appendix A for the methods and results). The scoping reviews relied on comprehensive search strategies applied in the following electronic databases (Medline, Global Health, and the WHO Library Database) and the grey literature (OpenGrey, DART-Europe and website searching). These reviews were complemented by the context-specific knowledge of the study team. A further 79 Secretariat and 22 country team/KTP indicators were identified from these sources. These indicators further expanded and populated the initial list of key indicators.

Third, the draft framework was peer-reviewed in two phases: (1) by international KT experts in April/May 2016 to ensure quality and feasibility. These experts were purposively selected and had both substantial experience in M&E and familiarity with EVIPNet. The framework was sent via email together with instructions and a peer-review template to be filled out. The refined framework was then (2) subsequently submitted to an expert online consultation. A maximum variation purposive sampling strategy was applied to select knowledgeable or experienced consultation participants and to ensure maximum variation in experience and responses, thus making more effective use of limited evaluation resources (Palinkas et al., 2015). Participants were found from the existing published and grey literature (OpenGrey, DART-Europe, and OAIster as well as hand searching through conference programmes), personal contacts (European Advisory Committee on Health Research, EVIPNet Europe national champions) and further snowball sampling. For the consultation, a total of eight experts participated on a pro bono basis (from 24 invited). The relatively low participation rate is a result of the fact that the consultation took place during the summer vacation period.

The data obtained were analysed by one member of the study team and discussed with a second team member to inform the revision of the framework. Discrepancies in interpretation were discussed and resolved. Key changes undertaken related to further detailing the ToC. Among other changes, the reviewers recommended including a causal logic for each of the domains for both the Secretariat and the country teams/KTPs, and to make the assumptions and contextual factors more explicit. Moreover, the sets of indicators were reworded for clarity and aligned with the amended ToCs and logic models. Also, to further guide the user, a column on the proposed “data collection methods" was added to the indicator tables and the sets of indicators were prioritized by creating two categories of ‘required’ and ‘recommended’ indicators. Indicators that are ‘required’ are those that in the context of EVIPNet Europe are essential for measuring the network’s progress and effectiveness at the Secretariat and country levels.

Finally, the peer-reviewed and amended framework was piloted during the EVIPNet Europe formative evaluation (Lester et al., 2020) to further develop it as a tool to be used by all stakeholders of EVIPNet Europe (and other KT initiatives) who are involved with promoting, strengthening and institutionalizing EIP at national and regional levels. A mixed methods design was utilized to pilot the framework. For the details on the process and results see (Lester et al., 2020). Following this pilot phase, the M&E framework was further revised by the team member who led the pilot through structured reflections and deliberations with the study lead. Each of the three domains at both WHO Secretariat and country team level were reviewed and revised in turn following this practical application. The wording of some of the indicators was refined to make them easier to measure and some data collection methods/sources that were identified during the piloting were added. In addition, gender equality measures/indicators (as suggested by the evaluation steering group review) were added. As a final step, the indicator tables were carefully reviewed and further simplified by streamlining and reducing the overall number of indicators, including removing any duplication.

## 4 Results

The framework outlines (i) a ToC (Brennan et al., 2017; ESSENCE on Health Research, 2016; Garforth, Ozor, Usher, & Bell, 2014; Hanley, Gould, Harle, & Nelson, 2012; Jones, 2011; Morton et al., 2012; Tsui, Simon, & John, 2014; Vogel, 2012; Vogel & Punton, 2017), and (ii) measures (indicators) to understand the extent to which EVIPNet Europe is implemented and its progress (both at the Secretariat and country team/KTP level). The indicators are separated into required (core) and recommended indicators. Some suggested data collection methods are also included with the indicators.

### 4.1 The EVIPNet Europe theory of change

**Fig. 4:**
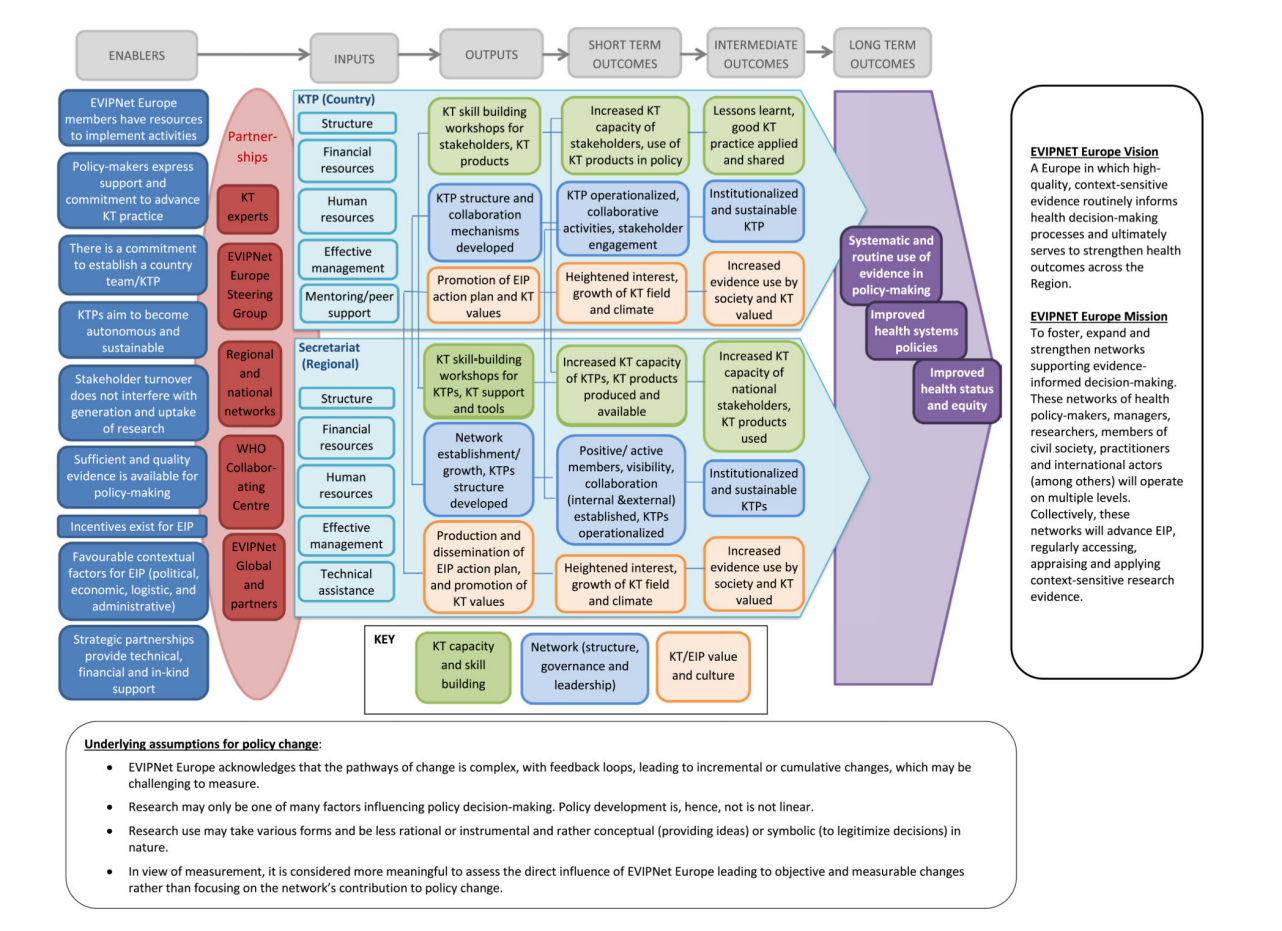
EVIPNet Europe Theory of Change. Source: Adapted from Lester et al. (2020).

A ToC describes “how the policy influencing activities are envisaged to result in the desired changes in policy or in people’s lives” (Jones, 2011, p. 3). The EVIPNet Europe ToC includes contextual factors (enablers) in which EVIPNet Europe member countries operate, an expected pathway of change and the underlying assumptions for policy change. Based on the EVIPNet Europe Strategic Plan (WHO Regional Office for Europe, 2015), changes need to be realized in three domains for the network to successfully implement its mandate: 1) KT capacity and skill building; 2) network (structure, governance and leadership); and 3) KT/EIP value and culture (see Fig. 4).

As noted in the methods section, the ToC of this framework relies on two seminal M&E approaches: 1) ‘outcome mapping’ (Earl et al., 2001), which is a methodology for monitoring and evaluating complex development processes such as KT (Balls & Nurova, 2020) and; 2) the logical framework (log frame) approach (Morton et al., 2012), one of the most used types of ToC, which describes a succession of elements and causal interlinkages between them (Jones, 2011).

At the programme level, the log frame sets out the hypothesized causal chain that can serve as a basis for a ToC. It identifies the assumptions, the required inputs (including resources and investments) that affect the immediate deliverables (outputs), leading to short- and intermediate changes (outcomes). Impacts (changes at a system and/or population level) are challenging to attribute to the programme activities since they occur a long way downstream from programme implementation and through the contributions of many players and forces (Earl et al., 2001). Therefore, although impact is included in the ToC, the M&E framework of EVIPNet Europe follows the ‘outcome mapping’ approach and focuses on changes occurring within a programme’s sphere of control (the network’s operational environment, i.e. inputs, activities and outputs) and influence (the network’s wider environment in which the network can effect change, i.e. outcomes) (Earl et al., 2001). Detailed logic models for both the WHO Secretariat and the country teams/KTPs can be found in Tables 1 and 2, respectively.

A range of contextual enablers were identified as an important component of the ToC. These can be seen on the left-hand side of Fig. 4. The process of joining EVIPNet Europe as a new network member includes assessing many of these contextual factors. Overall, these may vary over time and from country to country. The enablers include:

- EVIPNet Europe members have resources to implement activities;
- Support and commitment of policy-makers to promote KT;
- There is a commitment to establish a country team/KTP in an EVIPNet Europe member country;
- Country teams/KTPs aim to become autonomous and sustainable in implementing EVIPNet Europe activities;
- Stakeholder turnover does not jeopardize the production and use of evidence;
- Sufficient and quality evidence is available;
- Incentives exist for EIP;
- Favourable contextual factors for EIP (political, economic, logistic, and administrative); and
- Strategic partnerships provide technical, financial and in-kind support.

Jointly with its partnerships (shown in the second column of Fig. 4), EVIPNet Europe generates and provides the required inputs for its capacity building and KT activities. The inputs (shown in the third column of Fig. 4) include the network structure both at the network- and country-level, sufficient human resources (e.g. staff, consultants, fellows, interns, etc.), adequate financial resources (e.g. WHO core contributions and voluntary contributions to the network), effective management of resources and KT activities, including technical assistance of the WHO Secretariat, and mentoring and peer support.

EVIPNet Europe is meant to use these inputs to achieve the foreseen outputs and outcomes, grouped according to the three key domains of the ToC, i.e. 1) KT capacity- and skill-building; 2) Network (structure, governance and leadership); 3) KT/EIP value and culture. While the ToC provides an overview of the outputs and outcomes foreseen at both the WHO Secretariat and country levels, the logic models outlined in Tables 1 and 2 provide more detailed information.

There are a number of assumptions behind this ToC (summarized in Fig. 4), which would be necessary for EVIPNet Europe to achieve its objectives.

- EVIPNet Europe acknowledges that the pathway of change is complex, with feedback loops, leading to incremental or cumulative changes, which may be challenging to measure (Brennan et al., 2017; Jones, 2011).
- In KT processes, research plays a primary role but is not the only factor in policy decision-making. Research competes with factors such as local context and contingencies, different interests, priorities, ideas and values (Green & Bennett, 2007; Oliver et al., 2014; Verboom, Montgomery, & Bennett, 2016).
- Research use may take different forms and be less instrumental and rather conceptual in nature (to introduce new ideas and concepts to guide thinking and action) or symbolic (to e.g. legitimize decision-making) (Lavis et al., 2009; Weiss, 1979).
- Since it is challenging to assess the contributions of one programme/initiative to policy changes, it is advisable for EVIPNet Europe to concentrate its M&E efforts on assessing its direct influence (where the network’ s contributions are assumed) or where changes are measurable (Institute of Development Studies, 2013). These M&E efforts can also serve as the basis for an intervention to foster evidence-informed policies or active critical reflection on how and why EIP could be addressed (Tudisca et al., 2018).

### 4.2 Output and outcome indicators to measure performance of the WHO Secretariat and the country teams/KTPs

Based on the detailed logic models, sets of indicators were identified: 100 output and outcome indicators at WHO Secretariat level and 52 at country team/KTP level were included in the final framework. Tables 1 and 2 summarize the structure of M&E indicators in the developed logic model. Appendices B and C provide a menu of indicators for KT network secretariats and member countries respectively to choose from, depending on their own contexts, targets and expected changes.

To make the framework more manageable and provide further guidance to the users, the indicators were classified into ‘required’ or ‘recommended’ to facilitate their application (see Appendices A and B). They were also separated into output, short-term outcome and intermediate outcome indicators in line with the ToC and detailed logic models (Tables 1 and 2). The user can, thus, more easily identify the relevant indicators depending on the M&E purpose and network maturity. For example, if the network is still in a formative phase, the output and short-term outcome indicators will be most relevant.

Indicators that were labelled as ‘required’ are those that in the context of EVIPNet Europe are essential for measuring the network’s progress and effectiveness at the Secretariat and country levels. Other KT networks using the indicators may choose a different set of ‘required’ indicators, depending on their context. Some suggested data collection methods are also included with the indicators. While some of these are specific to the EVIPNet Europe context most can easily be adapted for other KT networks.

## 5 Discussion

As EVIPNet Europe continues to expand, a critical activity to its sustainability and success is assessing its work and current achievements to inform future developments and strategic planning of the network. EVIPNet Europe has therefore developed the comprehensive M&E framework to be used by the WHO Secretariat of EVIPNet Europe and its member countries. The framework may also serve other stakeholders involved with KT networks in their efforts to assess the performance, effectiveness and efficiency of their network’s approaches and activities. M&E is important because the science and practice of KT is growing rapidly, making it even more necessary to understand how best to translate knowledge into policy and practice (Ekirapa-Kiracho et al., 2014).

Based on the outcome mapping methodology, the EVIPNet Europe M&E framework focuses on changes in behaviours and relationships that can be linked to the network’s activities both at the WHO Secretariat and the country team/KTP levels. This is aligned with and promotes good practice in KT M&E (Balls & Nurova, 2020; Bennett & Jessani, 2011; Earl et al., 2001; Young et al., 2014). A ToC and the underlying log frame guide the user in their M&E efforts. However, the relevant indicators and data collection methods need to be selected dependent on the stage of network maturity, the specific KT context (enablers) and the assessment purpose (including whether the indicators are used for continuous monitoring of the programme or for evaluating the KT activities; both in a formative and summative manner).

It should be noted that a log frame, although much used in KT M&E (Bennett & Jessani, 2011; ESSENCE on Health Research, 2016; Garforth et al., 2014; Hanley et al., 2012; Jones, 2011; McLean & Tucker, 2013; The Networks of Centres of Excellence Secretariat, 2008; Tsui et al., 2014; Vogel & Punton, 2017; Yazdizadeh, Majdzadeh, Alami, & Amrolalaei, 2014), is a heuristic device aiming at simplifying complex and dynamic processes of how the policy influencing activities are meant to result in the foreseen changes in policy and practice (Asian Development Bank, 2010; Jones, 2011; Ottoson et al., 2009).

### 5.1 Lessons learned

A comprehensive framework was designed, with different levels and domains and a high number of quantitative and qualitative indicators. While the framework was initially difficult to navigate, feedback from the peer-reviewers and experience gained through the piloting helped us to contextualize the framework to provide further guidance for the user and to make it more user-friendly – without losing the framework’s intended comprehensiveness. Two important changes were made as a result of these steps: (i) the indicators were classified into required (core) and recommended indicators, thus providing further guidance to the users in selecting relevant indicators; and (ii) the indicator tables were carefully reviewed and further simplified by streamlining and reducing the overall number of indicators. An important lesson learned when piloting the framework was that ample time needs to be put aside to clarify the evaluation purpose, identify the related evaluation question(s), determine the data collection procedures, and in particular to identify appropriate indicators given the size of the draft M&E frame-work. Also, the timing of the data collection needs to be well chosen and vacation periods avoided, if possible, to ensure availability of respondents. Finally, setting-up and closely collaborating with a steering group – comprised of relevant stakeholders, evaluation experts and intended beneficiaries – to obtain advice when planning for and conducting the evaluation proved to be highly valuable in increasing the quality, utility and use of the evaluation work.

### 5.2 Strengths and limitations

The EVIPNet Europe M&E framework is one of few M&E frameworks that goes beyond assessing individual-level changes to also focus on the progress and performance at both an organizational- and system-level. It was developed based on a comprehensive methodology: literature reviews, expert review and consultation, as well as pilot-testing to ensure maximum relevance and feasibility of the framework and its indicators. This mixed-methods, iterative approach provided a solid foundation for the current version of the framework. A limitation of the process to date is that we were not able to pilot the full set of framework indicators due to time constraints and the current level of network maturity of EVIPNet Europe. Instead, we focused more on piloting the Secretariat’s logic model. To some degree, we tried to mitigate this effect through a careful process of selecting the indicators, guided by a M&E steering group. However, this means that some indicators of the final M&E framework have not yet been tested within the context of EVIPNet Europe for their suitability and feasibility. However, the EVIPNet Europe Framework should be seen as a living document that will be periodically revised and updated by the WHO Secretariat of EVIPNet Europe.

Another limitation of this, and any M&E framework, is that it does not guarantee that the results will be used to improve practice. As pointed out by Marra (Marra, 2021) cognitive biases associated with evidence use in decision-making hampers the instrumental use of evaluation findings. Marra suggests that one way to circumvent this may be to include a behavioural design into government and institution evaluation policies.

### 5.3 Conclusions and next steps

Establishing a framework with a ToC and indicators for both the Secretariat and the country teams/KTPs fills an important gap in the literature. It provides an instrument that addresses network evaluation holistically and that can be flexibly adapted and applied to the unit of analysis and stage of development. The EVIPNet Europe M&E framework is as an analytical tool providing technical guidance to stake-holders when developing specific KT evaluation plans at various stages of the maturity of a network/KT initiative.

The COVID-19 pandemic has reminded us, yet again, of the importance of mobilizing the best available evidence rapidly for sound and expeditious decision-making. To a large extent, this relies on existing KT capacity, mechanisms and infrastructure and their regular evaluation for quality improvements and learning (El-Jardali et al., 2020; Salvador-Carulla, Rosenberg, Mendoza, & Tabatabaei-Jafari, 2020; Tricco et al., 2020). While the framework has been successfully piloted (for the results of the formative evaluation (see (Lester et al., 2020)), a systematic user validation, in particular at country level, is still outstanding. It is envisaged that this will be undertaken to validate the amendments made based on the pilot testing (i.e. prioritization and streamlining of indicators) and to test the remainder of the indicators for feasibility.

To support network member countries in M&E, the development of a M&E guide and templates, training, and the establishment of a community of practice would be valuable. The WHO Secretariat should also consider repeating its evaluation in five years, in line with the EVIPNet Europe strategy. In addition, while aspects of KT institutionalization were included in the M&E framework, these could be further explored as the process for achieving, and determinants of, genuine sustainability and longevity of the network’s activities, both at regional and country levels, are not well-known.

## Supplementary Material

Supplementary data associated with this article can be found in the online version at doi:10.1016/j.evalprogplan.2022.102053.

Appendix A_Scoping review to identify indicators_Secretariat

Appendix_B_Manuscript KT framework_Secretariat indicators

Appendix_C_Manuscript KT framework_KTP indicators

## Figures and Tables

**Table 1:** EVIPNet Europe M&E Framework: WHO Secretariat level logic model.

Inputs	Outputs		Outcomes		Impact
			Short-term	Intermediate	
Structure Financial resources Human resources Effective management Technical assistance	**KT capacity and skill building**	**Capacity building for country teams/KTPs:** Skill-building workshops were convened by the WHO Secretariat on 1) KT skills (searching, accessing, appraising, synthesizing and using evidence) and 2) training facilitation and training-the-trainer skills **Technical support for KT products and activities:** Mentoring, coaching and reviews provided by the WHO Secretariat on situation analyses, EBPs, PDs and stakeholder meetings, rapid response services, clearinghouses, monitoring and evaluation and other KT activities at country level **WHO technical documents, tools and resources:** Guidance documents developed by the WHO Secretariat	**Increased KT capacity among country teams/KTPs through activities implemented by the WHO Secretariat:** 1) KT skills (searching, accessing, appraising, synthesizing and using evidence) and 2) training facilitation and training-the-trainer skills were gained through skill-building workshops **Increased KT practice and production/availability of KT products by country teams/KTPs:** EBPs, PDs, etc. were produced at national and regional levels by applying skills gained through capacity building activities	**Increased KT capacity among external stakeholders** through activities implemented by country teams/KTPs (searching, accessing, appraising, synthesizing and using evidence) **Use of KT products by country teams/KTPs and external stakeholders:** KT products created by workshop participants were used at national and regional levels	Systematic and routine use of evidence in policymaking Improved health system policies Improved health status and equity
	**Network (structure, governance and leadership)**	**Network membership:** Incentives for joining the network created by the WHO Secretariat and network established at country level **Strategic directions and activities:** Strategy and a vision for the network developed by the WHO Secretariat, aligned with country needs **Network governance:** Network/resource management and leadership mechanisms created by the WHO Secretariat	**Network membership growth and strengthening:** Network growth and stability **Internal and external visibility:** EVIPNet Europe seen as an expert in the KT field, adding value **Country teams/KTPs established and operationalized:** EVIPNet Europe established and KT work initiated	**Network member satisfaction:** Member countries satisfied with EVIPNet Europe and its activities **Demonstration of efficiency and effectiveness in KT:** EVIPNet Europe showed to be efficient and effective through its KT activities **Institutionalization of sustainable KTPs:** National and regional networks becoming self-sustaining	
		**Mechanisms for exchange and networking:** A system for sharing information and networking both internally and externally created by the WHO Secretariat	**Strengthened collaboration and partnerships:** EVIPNet Europe established collaborative projects, partnerships and networking opportunities, both network-internal and external		
	**KT/EIP value and culture**	**Production and dissemination of and EIP action plan and promotion of KT values:** Publication of an action plan to strengthen the use of evidence, information and research for policy-making in the WHO European Region	**Increased commitment to KT:** Heightened interest, political support, growth of KT field and climate	**Increased evidence us by society and KT values:** Evidence use increasingly mainstreamed through society	

**Table 2:** EVIPNet Europe M&E Framework: Country team/KTP level logic model.

Inputs	Outputs		Outcomes		Impact
			Short-term	Intermediate	
Structure Financial resources Human resources Effective management Technical assistance	**KT capacity and skill building**	**Capacity building for country teams/Knowledge Translation Platforms (KTP) and external stakeholders convened by the Evidence-informed Policy Network (EVIPNet) Europe country teams/KTPs:**1) Skill-building workshops on knowledge translation (KT) and training facilitation skills attended by the country team/KTP and 2) KT skills building workshops for external national stakeholders organized by the country team/KTP	**Increased KT capacity:** Increased KT knowledge and skills of country teams/Knowledge Translation Platforms (KTP) and external stakeholders to search/access/appraise/synthesize/use research evidence	**Increased use of KT products** at national and Regional levels	Systematic and routine use of evidence in policymaking Improved health system policies Improved health status and equity
		**KT products and activities developed by country/teams/KTP:** Health policy topic issues prioritized; evidence briefs for policy (EBPs) on priority health issues developed; policy dialogues (PDs) organized	**Increased production and visibility of KT products** produced by KTP, searchable and discoverable		
		**Monitoring and evaluation (M&E) plan:** Mechanism for regular M&E in place	**Routine M&E** by the country team/KTP	**Improved country team/KTP performance:** Lessons learnt and good practice used, applied and shared by KTP for improving KTP and overall network operationalization	
	**Network (structure, governance and leadership)**	**Country team/KTP engaged with EVIPNet Europe:** Situation analysis (SA) conducted, stakeholder meetings held **Country team/KTP structure developed:** Resources, Governance structure put in place, management and leadership, A formal/informal country team/KTP is established	**Operationalization of KTP:** Country teams plan, implement, monitor and evaluate KT activities in support of EIP	**Institutionalization of sustainable KTP:** Regional and national networks are self-sustainable and autonomous	
		**Mechanisms for collaboration and peer-exchange** established	**Production of collaborative activities and stakeholder engagement** by the country team/KTP		
	**KT/EIP value and culture**	**Promotion of EIP action plan and KT values:** KT agenda setting and promotion through the EIP action plan	**Increased commitment to KT:** Heightened interest, growth of KT field and climate	**Increased evidence us by society and KT values:** Evidence use increasingly mainstreamed through society	

## Footnotes

1 KTPs bring decision-makers, researchers, practitioners, civil society groups and other stakeholders together to facilitate the process of translating evidence into policy and action by: aligning research topics with policy priorities, responding to pressing issues through developing policy briefs, rapid responses and evidence summaries; and convening dialogues to guide policy formulation and implementation, taking into consideration local and political context.

## Abbreviations

EIP,: evidence-informed policy-making
EVIPNet,: Evidence-informed Policy Network
EBP,: evidence brief for policy
KT,: knowledge translation
KTP,: Knowledge Translation Platforms
M&E,: monitoring and evaluation
PD,: policy dialogue
SA,: Situation analysis
ToC,: Theory of change
WHO,: World Health Organization
