# Supplementary material for: A comprehensive monitoring and evaluation framework for evidence to policy networks
Source: Eval Program Plann. Author manuscript; Available in PMC 2023 Jan 12. (PMC7614046; doi:10.1016/j.evalprogplan.2022.102053)
Supplement: Appendix A_Scoping review to identify indicators_Secretariat [file EMS159051-supplement-Appendix_A_Scoping_review_to_identify_indicators_Secretariat.pdf]

**Appendix A**

## Scoping review to identify indicators to measure the work of the WHO Secretariat of EVIPNet Europe

Even though EVIPNet Europe Secretariat is a type of KTP, it has additional functions as a coordinating body of several nodes. Given that the first mapping exercise focused on country-level/KTP indicators, it did not cover all relevant aspects of a secretariat body. Thus, in order to collect indicators that more accurately represent the secretariat, a second indicator mapping exercise focused on secretariats was conducted.

### Methods

Data collection: Data was iteratively collected in a four-step approach, including two peer-literature database reviews, a grey literature database review, and a website review of similar secretariat organizations to EVIPNet Europe. Collection began with step 1, but since no results were found we proceeded with the additional three steps.

**Step 1: Peer-literature database review searched in Medline and Global Health databases**

Following on from the scoping review conducted by Scarlet and colleagues[1] for the KTP indicator mapping exercise, a similar methodology was used. A search strategy was drafted, piloted, and refined in consultation with the librarian staff at Karolinska Institutet, as well as review by consultant Tarang Sharma from Cochrane (Annex 2). The search strategy used by Scarlett and colleagues was revised to account for the different secretariat, perspective and based on their experience from the screening process.

Articles were eligible for inclusion if they were (1) published from 2005-2017; (2) in English; (3) focused on the evaluation of a secretariat or the coordination of KT between research and policy by a secretariat; and (4) included specific indicators for measurement.

**Step 2: Peer-literature database review searched in Medline and Global Health databases with different eligibility criteria**

The results from Step 1 were re-evaluated to see if a change in eligibility criteria resulted in more eligible articles. All eligibility criteria remained unchanged, except for "the coordination of KT between research and policy" which was changed to "the coordination of KT between research and policy or practice".

**Step 3: New search using grey literature databases OpenGrey, DART-Europe, and OAIster**

A new search strategy was developed for grey literature databases (Annex 3). Eligibility criteria were the same as those used in step 1.

Note: The OAIster search had to be stopped, since it is a search engine and not a database, so it was impossible to screen articles systematically.

**Step 4: Website search of similar 'secretariat' organizations involved in coordinating KT at several nodes**

Since no relevant results were found in the systematic searches, the websites of similar secretariat organizations to EVIPNet Europe were hand searched.

---

[1] Scarlett, J., Forsberg, B. C., Biermann, O., Kuchenmüller, T., & El-Khatib, Z. (2020). Indicators to evaluate organisational knowledge brokers: a scoping review. *Health Res Policy Syst, 18*(1), 93. doi:10.1186/s12961-020-00607-8.

Data sources included the Bridge report, previous knowledge brokering mapping, and sources identified by supervisor or google searching.

Organizations were eligible for inclusion if they were a secretariat organization (defined as an organization coordinating several nodes) that funds, provides technical support, coordinates capacity building or network development for KT.

Websites were searched for evaluations (frameworks, final or midterm reports) or organizational strategies, as these sometimes included indicators to measure success.

## Results

**Step 1: Peer-literature database review searched in Medline and Global Health databases**
Total, duplicates removed = 477
Abstract screened, excluded = 438*
*(not: secretariat, policy, KT, evaluation, capacity building; no access)
Full text screened 39
Final count for analysis = 0

**Step 2: Peer-literature database review searched in Medline and Global Health databases with different eligibility criteria**
Total items re-screened = 38
Excluded = 38 (not: secretariat, evaluation)
Final count for analysis = 0

**Step 3: New search using grey literature databases OpenGrey, DART-Europe, and OAIster**
Total items screened: 20
Excluded: 20 (not: secretariat, policy, KT, evaluation, capacity building)
Final count for analysis = 0

**Step 4: Website search of similar 'secretariat' organizations involved in coordinating KT at several nodes**
35 organizations (Annex 4)
47 documents extracted
Excluded: 37 excluded (not output/outcome evaluation, no indicators)
Final count for analysis = 13 (Annex 5)

## Annex 1: KTP indicator mapping search strategy

1. exp "Diffusion of Innovation"/

2. exp Information Dissemination/

3. ((knowledge or evidence-based or research*) adj2 (action or collaborat* or implement* or adopt* or applicat* or broker* or coproduction or diffusion or dissemination or exchang* or mobilization or mobilisation or network* or platform* or shar* or use* or using or synthes* or transfer* or translat* or uptake or utilization or utilisation)).ti,ab.

4. (evidence informed or knowledge intermediar* or "linkage and exchange").ti,ab.

5. 1 or 2 or 3 or 4

6. exp Decision Making/

7. exp Decision Making, Organizational/

8. exp Policy Making/

9. exp Health Planning Organizations/

10. (Decision mak* or Health systems strengthening or Policy* or Policies).ti,ab.

11. 6 or 7 or 8 or 9 or 10

12. exp Evaluation Studies/

13. exp Program Evaluation/

14. exp Capacity Building/

15. ((change* or measur* or monitor* or improv* or increas* or evaluat* or build* or achiev*) adj3 (capacity or evidence-informed or knowledge translation or knowledge use* or evidence use* or research use*)).ti,ab.

16. 12 or 13 or 14 or 15

17. 5 and 11 and 16

## Annex 2: secretariat indicator mapping search strategy

Search string (OVID)

1. exp Diffusion of Innovation/

2. exp Information Dissemination/

3. knowledge adj2 (action or collaborat* or implement* or applicat* or broker* or coproduction or diffusion or disseminat* or exchang* or intermediary or mobilization or mobilisation or network* or platform* or shar* or use* or using or synthes* or transfer* or translat* or uptake or utilization or utilisation)).ti,ab.

4. research adj2 (policy* or policies).ti,ab.

5. (evidence informed).ti,ab.

6. 1 or 2 or 3 or 4 or 5

7. exp Decision Making/

8. exp Decision Making, Organizational/

9. exp Policy Making/

11. (Decision mak* or Health systems strengthening or Policy* or Policies).ti,ab.

12. 7 or 8 or 9 or 10 or 11

13. exp Evaluation Studies/

14. exp Program Evaluation/

15. (change* or measur* or monitor* or improv* or increas* or evaluat* or build* or achiev*).ti,ab.

16. 13 or 14 or 15

17. exp Health Planning Organizations/

18. exp Capacity Building/

19. (secretaria* or capacity building organization* or change agent* or agent* of change or donor or donors or funder or funders).ti,ab.

20. stakeholders adj2 (empower* or engag* or conven* or support* or coordinat*).ti,ab.

21. Capacity adj2 (build* or develop* or local).ti,ab.

22. 17 or 18 or 19 or 20 or 21

23. 6 AND 12 AND 16 AND 22

24. limit 21 to (abstracts and English language and humans and yr= “2005-2016”)

## Annex 3: Search terms used in grey literature databases

"knowledge transfer"

"knowledge translation"

"evidence-informed policy"

"capacity building"

coordinating OR coordinated OR coordinate

engage OR engaging OR engagement

support OR supporting OR supported

funders or funder or funded

evaluation OR evaluated OR evaluate OR evaluating

## Annex 4: Secretariat organizations collected for searching

| Institution | Website |
|---|---|
| 1. EVIPNet | http://www.who.int/evidence/en/ |
| 2. International Network for the Availability of Scientific Publications (INASP) | http://www.inasp.info/en/ |
| 3. BCURE | https://bcureglobal.wordpress.com/ |
| 4. Knowledge Translation Canada | http://ktcanada.org/ |
| 5. Canadian Institutes of Health Research | http://www.cihr-irsc.gc.ca/e/193.html |
| 6. Canadian Academy of Health Sciences/Académie canadienne des sciences de la santé | https://cahs-acss.ca/ |
| 7. National Collaborating Centre for Public Health (Methods & Tools) | http://www.nccmt.ca/<br><br>http://nccph.ca/ |
| 8. Networks of Centres of Excellence of Canada | http://www.nce-rce.gc.ca/Index_eng.asp |
| 9. EuroHealth Net | http://eurohealthnet.eu/ |
| Regions for Health Network | http://www.euro.who.int/en/about-us/networks/regions-for-health-network-rhn |
| 10. National Institute for Health and Welfare Finland | https://www.thl.fi/en/web/thlfi-en |
| 11. Alliance for Health Policy and Systems Research | http://www.who.int/alliance-hpsr/en/ |
| 12. Joanna Briggs Institute | http://joannabriggs.org/ |
| 13. Global Development Network | http://www.gdn.int/ |
| 14. Evidence Based Policy in Development Network | https://partnerplatform.org/ebpdn/ |
| 15. Department of International Development | https://www.gov.uk/government/organisations/department-for-international-development |
| 16. International Development Research Centre | https://www.idrc.ca/ |
| 17. HENVINET | http://www.henvinet.eu/ |
| 18. FUSE | http://www.fuse.ac.uk/ |
| 19. Knowledge exchange | http://www.knowledge-exchange.info/ |
| 20. Cochrane | https://www.cochrane.org/ |

| | |
|---|---|
| 21. Measure Evaluation | https://www.measureevaluation.org/about |
| 22. fhi360 | https://www.fhi360.org/about-us/vision-and-mission |
| 23. Food and Agriculture Organization of the United Nations | http://www.fao.org/in-action/search-results/en/?cx=018170620143701104933%3Aqq82jsfba7w&q=knowledge+translation&cof=FORID%3A9&siteurl=www.fao.org%2Fin-action%2Fen%2F&ref=www.fao.org%2Fabout%2Fhow-we-work%2Fen%2F&ss=2064j461696j14 |
| 24. 3ie International Initiative for Impact Evaluation | http://www.3ieimpact.org/ |
| 25. Counterpart International | http://www.counterpart.org/ |
| 26. International Centre for Policy Advocacy | http://www.icpolicyadvocacy.org/about-icpa |
| 27. International Organization for Cooperation in Evaluation | https://www.ioce.net/ |
| 28. Joint Learning Network | http://www.jointlearningnetwork.org/ |
| 29. On Think Tanks | https://onthinktanks.org/ |
| 30. REPOPA (Research into Policy to Enhance Physical Activity) project | http://www.repopa.eu/ |
| 31. Strengthening Research Capacity and Evidence-Based Policy-Making in Afghanistan and Central Asia: A Research and Public Policy Initiative | https://idl-bnc-idrc.dspacedirect.org/handle/10625/56356 |
| 32. The European Implementation Collaborative | https://www.implementation.eu/ |
| 33. The Stockholm Institute of Transition Economics, Stockholm School of Economics | https://www.hhs.se/en/research/institutes/site/ |
| 34. European Cluster Collaboration Platform | https://www.clustercollaboration.eu/vibrant-platform-service-cluster-organisations |
| Non institution literature (manually added by TK) | https://www.iisd.org/pdf/2004/networks_guidelines_for_assessment.pdf |
| Non institution literature (manually added by TK) | Network Maturity Matrix: https://www.source4networks.org.uk/images/site/files/Maturity_Model_Matrix_v2_071216-FINAL.pdf |

## Annex 5: References of included papers or reports


1. Chapman, E. (2012). Evaluation of the Evidence Informed Policy Networks (EVIPNet). Washington, D.C.: PAHO.

2. Creech, H., & Ramji, A. (2004). Knowledge Networks: Guidelines for Assessment. Winnipeg, Manitoba: International Institute for Sustainable Development.

3. European Implementation Collaborative. (n. d.). EIC Logic Model. In: European Implementation Collaborative

4. Global Development Network. (2017). The Road Ahead. Strategy 2017-2022. Local Research for Better Lives. In. Washington, D.C.: GDN.

5. Hanley, T., Gould, C., Harle, J., & Nelson, K. (2012). International Network for the Availability of Scientific Publications. Programme for the Enhancement of Research Information. Phase II. External Evaluation 2008-12. Final Report. Oxford.

6. The Joanna Briggs Institute. (2015). 2016 - 2020 Strategic Plan. Better evidence. Better outcomes. Brighter future. Taking JBI to 2020 and beyond… Adelaide: The University of Adelaide.

7. McLean, R., & Tucker, J. (2013). Evaluation of CIHR's Knowledge Translation Funding Program. Ottawa: Canadian Institutes of Health Research.

8. Morton, J., Shaxson, L., & Greenland, J. (2012). Final Report. Process Evaluation of the International Initiative for Impact Evaluation (2008-11). London: Triple Line Consulting Ltd/Overseas Development Institute.

9. Panel on Return on Investment in Health Research. (2009). Making an Impact. A Preferred Framework and Indicators to Measure Returns on Investment in Health Research. (C. A. o. H. Sciences Ed.). Ottawa, ON: Canadian Academy of Health Sciences.

10. Sources4Network. (2016). Network Maturity Matrix. In. London: NHS England [website]. https://www.source4networks.org.uk/images/site/files/Maturity_Model_Matrix_v2_071216-FINAL.pdf.

11. The Networks of Centres of Excellence Secretariat. (2008). Joint Results-based Management and Accountability Framework and Risk-Based Audit Framework for the Class Grant Networks of Centres of Excellence Program. Ottawa: The Networks of Centres of Excellence Secretariat.

12. Vogel, I., & Punton, M. (2016). Building Capacity to Use Ressearch Evidence (BCURE) Evaluation: Stage 1 Synthesis Report. Brighton: Itad.

13. Vogel, I., & Punton, M. (2017). Building Capacity to Use Research Evaluation (BCURE) realist evaluation: Stage 2 Synthesis Report: ITAD.
