# Supplementary material for: A comprehensive monitoring and evaluation framework for evidence to policy networks
Source: Eval Program Plann. Author manuscript; Available in PMC 2023 Jan 12. (PMC7614046; doi:10.1016/j.evalprogplan.2022.102053)
Supplement: Appendix_B_Manuscript KT framework_Secretariat indicators [file EMS159051-supplement-Appendix_B_Manuscript_KT_framework_Secretariat_indicators.pdf]

Appendix B. EVIPNet Europe M&E framework: Secretariat indicators

(I) KT capacity and skill building

OUTPUTS	INDICATORS*	DATA COLLECTION METHODS
**Capacity building for country teams/KTPs:** Skill-building workshops were convened by the WHO Secretariat on 1) KT skills (searching, accessing, appraising, synthesizing and using evidence) and 2) training facilitation and training-the-trainer skills	• **#/types of KT skill-building workshops for country teams/KTPs** (Chapman, 2012; Ongolo-Zogo, Lavis, Tomson, & Sewankambo, 2014; Ottoson et al., 2009; The Networks of Centres of Excellence Secretariat, 2008; Waqa, Mavoa, Snowdon, Moodie, Schultz, et al., 2013)<br>• **# of countries participated in KT skills building workshops for country teams/KTPs** (Chapman, 2012)<br>• # of country team/KTP participants and their affiliations (Chapman, 2012; de-Graft Aikins et al., 2012; Ongolo-Zogo et al., 2014; Waqa, Mavoa, Snowdon, Moodie, Schultz, et al., 2013) by gender (ESSENCE on Health Research, 2016; C.J.; Uneke, Ezeoha, Ndukwe, Oyibo, & Onwe, 2011; C. J. Uneke et al., 2015a, 2015b; Waqa, Mavoa, Snowdon, Moodie, Schultz, et al., 2013)<br>• Country team/KTP participant satisfaction scores from WHO organised KT skills building workshops (Neves, Lavis, Panisset, & Klint, 2014; C. J. Uneke et al., 2015b)	• Evidence-informed Policy Network (EVIPNet) Europe annual reports, performance tracking sheet, workshop evaluation report, WHO travel reports, WHO reporting<br>• Training feedback forms, qualitative interviews
**Technical support for KT products and activities:** Mentoring, coaching and reviews provided by the WHO Secretariat on situation analyses, EBPs, PDs and stakeholder meetings, rapid response services, clearinghouses, monitoring and evaluation and other KT activities at country level	• **#/type/topic of evidence products/activity developed by the country teams/KTPs supported by WHO Secretariat** (Chapman, 2012; Cole, Nyirenda, Fazal, & Bates, 2016; Ekirapa-Kiracho et al., 2014 ; ESSENCE on Health Research, 2016; Johnson & Lavis, 2010; Ongolo-Zogo et al., 2014)<br>• # of member countries that received individual mentoring from WHO Secretariat (Vogel & Punton, 2016)	• EVIPNet Europe annual reports, WHO reporting, published EBPs, PD reports, clearinghouses, rapid responses, stakeholder meeting reports, M&E reports, country team/KTP Annual Activities and Outputs Inventory
**WHO technical documents, tools and resources:** Guidance documents developed by the WHO Secretariat	• **#/types/topics of technical documents/tools/resources/training modules developed for country teams/KTPs by WHO Secretariat** (Global Development Network, 2017; Ottoson et al., 2009; Sources4Network; Vogel & Punton, 2016; Waqa, Mavoa, Snowdon, Moodie, Schultz, et al., 2013)	• EVIPNet Europe annual reports, WHO reporting, published tools

* Indicators **in bold are required**, others are recommended. Indicators without reference were recommended by EE stakeholders and/or the authors.

OUTCOMES (short-term)	INDICATORS*	DATA COLLECTION METHODS
**Increased KT capacity among country teams/KTPs through activities implemented by the WHO Secretariat:** 1) KT skills (searching, accessing, appraising, synthesizing and using evidence) and 2) training facilitation and training-the-trainer skills were gained through skill-building workshops	• **% increase in KT skills of workshop participants: Change from pre-post assessment scores** (Bartlett & Peach, 2008; Johnson & Lavis, 2010; Mavoa et al., 2012; C.J.; Uneke et al., 2011; C. J. Uneke et al., 2015a, 2015b)<br>• #/% of workshop participants who intend to use skills gained (Neves et al., 2014; Sallis et al., 2009)	• Workshop evaluation reports. training feedback forms (initial and 6-12 month follow up), qualitative interviews
	• % of participants that report a benefit of training (Bartlett & Peach, 2008; Morton, Shaxson, & Greenland, 2012; Neves et al., 2014; Sallis et al., 2009; C.J.; Uneke et al., 2011; C. J. Uneke et al., 2015a, 2015b; Waqa, Mavoa, Snowdon, Moodie, Nadakuitavuki, et al., 2013), by gender (ESSENCE on Health Research, 2016; C.J.; Uneke et al., 2011; C. J. Uneke et al., 2015a, 2015b; Waqa, Mavoa, Snowdon, Moodie, Schultz, et al., 2013)<br>- new and/or improved knowledge (Hanley, Gould, Harle, & Nelson, 2012; Hawkes et al., 2016; Neves et al., 2014; Sallis et al., 2009; C.J.; Uneke et al., 2011; C. J. Uneke et al., 2015a, 2015b; Vogel & Punton, 2016, 2017)<br>- new and/or improved skills (Hanley et al., 2012; Hawkes et al., 2016; Neves et al., 2014; Ottoson et al., 2009; C.J.; Uneke et al., 2011; C. J. Uneke et al., 2015a, 2015b; Vogel & Punton, 2016, 2017)<br>- shared experiences/lessons learnt (Neves et al., 2014)<br>- opportunities for collaboration (Neves et al., 2014; Sallis et al., 2009)<br>- renewed motivation (Neves et al., 2014)<br>- clarification of priority needs (Araujo de Carvalho et al., 2015; Neves et al., 2014)<br>• **#/% of workshop participants who intend to use skills gained** (Neves et al., 2014; Sallis et al., 2009), by gender (ESSENCE on Health Research, 2016; C.J.; Uneke et al., 2011; C. J. Uneke et al., 2015a, 2015b; Waqa, Mavoa, Snowdon, Moodie, Schultz, et al., 2013)<br>• # of workshop participants who intend to participate in future workshops	• Workshop evaluation reports, training feedback forms (initial and 6-12 month follow up), qualitative interviews

* Indicators **in bold are required**, others are recommended. Indicators without reference were recommended by EE stakeholders and/or the authors.

	(Bartlett & Peach, 2008; Neves et al., 2014; C.J.; Uneke et al., 2011; C. J. Uneke et al., 2015a, 2015b), by gender (ESSENCE on Health Research, 2016; C.J.; Uneke et al., 2011; C. J. Uneke et al., 2015a, 2015b; Waqa, Mavoa, Snowdon, Moodie, Schultz, et al., 2013)	
**Increased KT practice and production/availability of KT products** by country teams/KTPs: EBPs, PDs, etc. were produced at national and regional levels by applying skills gained through capacity building activities	• **#/types/topics of KT products developed by country teams/KTPs [per year]** (Chapman, 2012; Global Development Network, 2017)<br>• **#/types/topics of KT activities undertaken** (e.g. policy dialogues organized) **by country teams/KTPs** (Makkar et al., 2016; Vogel & Punton, 2016)<br>• **# of KT products/activities co-produced by country teams/KTPs with policy-makers** (Hawkes et al., 2016; The Networks of Centres of Excellence Secretariat, 2008)<br>• **# of views/downloads of tools and publications from the WHO/Europe EVIPNet Europe website** (Alberta Addiction and Mental Health Research Partnership Program, 2014; Ekirapa-Kiracho et al., 2014 ; Galluzzo et al., 2012; Peirson, Catallo, & Chera, 2013)<br>• Relevance to (Ekirapa-Kiracho et al., 2014 ; R. Makkar et al., 2016) and timeliness (Creech & Ramji, 2004) of EBPs in priority health issues	• Performance tracking sheet, EVIPNet Europe annual reports, WHO reporting, country team/KTP annual activities and outputs inventory<br>• Website tracking<br>• Qualitative interviews with policy-makers, Online evaluation (survey)

OUTCOMES (intermediate)	INDICATORS*	DATA COLLECTION METHODS
**Increased KT capacity among external stakeholders** through activities implemented by country teams/KTPs (searching, accessing, appraising, synthesizing and using evidence)	• **#/types of KT skill-building workshops for external stakeholders organised by country teams/KTPs** (Chapman, 2012; Ongolo-Zogo et al., 2014; Ottoson et al., 2009; The Networks of Centres of Excellence Secretariat, 2008; Waqa, Mavoa, Snowdon, Moodie, Schultz, et al., 2013)<br>• **# of external stakeholder participants and their affiliations** (Chapman, 2012; Ongolo-Zogo et al., 2014; Waqa, Mavoa, Snowdon, Moodie, Schultz, et al., 2013), by gender (ESSENCE on Health Research, 2016; C.J.; Uneke et al., 2011; C. J. Uneke et al., 2015a, 2015b; Waqa, Mavoa, Snowdon, Moodie, Schultz, et al., 2013)<br>• **% of participants that report a benefit of training** (Bartlett & Peach, 2008; Neves et al., 2014; Sallis et al., 2009; C.J.; Uneke et al., 2011; C. J. Uneke et al., 2015a, 2015b), by gender (ESSENCE on Health Research,	• Workshop evaluation reports, training feedback forms (initial and 6-12 month follow up), qualitative interviews

* Indicators **in bold are required**, others are recommended. Indicators without reference were recommended by EE stakeholders and/or the authors.

	2016; C.J.; Uneke et al., 2011; C. J. Uneke et al., 2015a, 2015b; Waqa, Mavoa, Snowdon, Moodie, Schultz, et al., 2013)- new and/or improved knowledge (Hanley et al., 2012; Hawkes et al., 2016; Neves et al., 2014; Sallis et al., 2009; C.J.; Uneke et al., 2011; C. J. Uneke et al., 2015a, 2015b; Vogel & Punton, 2016, 2017)<br>- new and/or improved skills (Hanley et al., 2012; Hawkes et al., 2016; Neves et al., 2014; Ottoson et al., 2009; C.J.; Uneke et al., 2011; C. J. Uneke et al., 2015a, 2015b; Vogel & Punton, 2016, 2017)<br>- shared experiences/lessons learnt (Neves et al., 2014)<br>- opportunities for collaboration (Neves et al., 2014; Sallis et al., 2009)<br>- renewed motivation (Neves et al., 2014)<br>- clarification of priority needs (Araujo de Carvalho et al., 2015; Neves et al., 2014)<br>• **#/% of stakeholders who intend to use skills gained** (Neves et al., 2014; Sallis et al., 2009), by gender (ESSENCE on Health Research, 2016)<br>• # of external stakeholders who intent to participate in future workshops (Bartlett & Peach, 2008; Neves et al., 2014; C.J.; Uneke et al., 2011; C. J. Uneke et al., 2015a, 2015b), by gender (ESSENCE on Health Research, 2016; C.J.; Uneke et al., 2011; C. J. Uneke et al., 2015a, 2015b; Waqa, Mavoa, Snowdon, Moodie, Schultz, et al., 2013)<br>• **# of external stakeholders who request training support from KTPs** (Vogel & Punton, 2017)	• Country team/KTP annual activities and outputs inventory
**Use of KT products by country teams/KTPs and external stakeholders:** KT products created by workshop participants were used at national and regional levels	• **# of citations of KT products (in policy, research and/or media documents, in speeches, debates and informal internal documents)** (Angulo-Tuesta, Santos, & Natalizi, 2016; Mahmood, Hort, Ahmed, Salam, & Cravioto, 2011; Panel on Return on Investment in Health Research, 2009; Vogel & Punton, 2016)<br><br>•**Reported use** (e.g. evidence presented and/or discussed in meetings, included as a technical document in policy formulation meetings, contributing to decision-making etc.) (Araujo de Carvalho et al., 2015; Ekirapa-Kiracho et al., 2014 ; Kwan et al., 2007; Mahmood et al., 2011; Murnaghan et al., 2013; Ottoson et al., 2009; Panel on Return on	• Website tracking and social network analysis<br><br>• Qualitative interviews, EVIPNet Europe annual reports, online evaluation (survey), country success stories

* Indicators **in bold are required**, others are recommended. Indicators without reference were recommended by EE stakeholders and/or the authors.

	Investment in Health Research, 2009; The Networks of Centres of Excellence Secretariat, 2008)	

(II) Network (structure, governance, and leadership)

OUTPUTS	INDICATORS*	DATA COLLECTION METHODS
**Network membership:** Incentives for joining the network created by the WHO Secretariat and network established at country level	• **# of EVIPNet Europe member countries** (The Networks of Centres of Excellence Secretariat, 2008)<br>• **# of EVIPNet Europe members and their affiliations** (de-Graft Aikins et al., 2012), by gender (ESSENCE on Health Research, 2016; C.J.; Uneke et al., 2011; C. J. Uneke et al., 2015a, 2015b; Waqa, Mavoa, Snowdon, Moodie, Schultz, et al., 2013)<br>• # of success stories in advocacy at the organizational, national, and international levels (Hanley et al., 2012)<br>• Growth strategy developed by the WHO Secretariat (The Joanna Briggs Institute, 2015)	• EVIPNet Europe annual reports, WHO reporting, WHO website<br>• EVIPNet Europe annual reports, WHO reporting, published tools
**Strategic directions and activities:** Strategy and a vision for the network developed by the WHO Secretariat, aligned with country needs	• **# of situation analyses conducted by member countries** to identify needs for EIP capacity building and institutionalization (Araujo de Carvalho et al., 2015; Hanley et al., 2012; McLean & Tucker, 2013; The Networks of Centres of Excellence Secretariat, 2008; Vogel & Punton, 2016, 2017; Waqa, Mavoa, Snowdon, Moodie, Schultz, et al., 2013)<br>• # of member countries that assessed KT/EIP capacity using ORACLe, SAGE and IRWFY tools (Mavoa et al., 2012; The CIPHER Investigators, 2014; Waqa, Mavoa, Snowdon, Moodie, Nadakuitavuki, et al., 2013)<br>• **% of alignment of EVIPNet Europe KT strategy with theory and empirical evidence of KT success strategies** (McLean & Tucker, 2013)<br>• **EVIPNet Europe strategy available stating innovation and learning as objective** (Hanley et al., 2012)<br>• **EVIPNet Europe's activities developed based on government priorities and country needs** (Hanley et al., 2012; The Networks of Centres of Excellence Secretariat, 2008; Waqa, Mavoa, Snowdon, Moodie, Schultz, et al., 2013)	• EVIPNet Europe annual reports, WHO reporting, WHO website, performance tracking sheet, EVIPNet Europe Multicountry Meeting reports, country team/KTP annual activities and outputs inventory<br>• EVIPNet Europe Strategic plan<br>• EVIPNet Europe operational plan

* Indicators **in bold are required**, others are recommended. Indicators without reference were recommended by EE stakeholders and/or the authors.

	• **Annual review process as part of the annual strategic planning session in place** (The Joanna Briggs Institute, 2015)	
**Network governance:** Network/resource management and leadership mechanisms created by the WHO Secretariat	• **# staff members in the WHO Secretariat, per gender** (Panel on Return on Investment in Health Research, 2009)<br>• **Stable staffing available to the network** (Ottoson et al., 2009)<br>• **Credible leader with dedicated time available** (Makkar et al., 2016; Sources4Network, 2016)<br>• Succession plan for leadership (Creech & Ramji, 2004)<br>• **Lists of members and organizational affiliation of the network steering group** (The Networks of Centres of Excellence Secretariat, 2008) **and # of meetings** (Vogel & Punton, 2016)<br>• **M&E framework published by WHO Secretariat** (The Joanna Briggs Institute, 2015)<br>• Availability of adequate human resources (skills, skill-building opportunities, KT stakeholder representation) (R. Makkar et al., 2016; The Networks of Centres of Excellence Secretariat, 2008; Waqa, Mavoa, Snowdon, Moodie, Nadakuitavuki, et al., 2013)<br>• Availability of adequate financial resources (sustainable funding, financial management) (Creech & Ramji, 2004)	• EVIPNet Europe Annual reports, WHO reporting<br>• EVIPNet Europe steering group minutes<br>• EVIPNet Europe Annual reports, WHO reporting, WHO website tracking<br>• EVIPNet Europe Annual reports, WHO reporting
**Mechanisms for exchange and networking:** A system for sharing information and networking both internally and externally created by the WHO Secretariat	• **Availability of functional virtual platforms for peer support and sharing lessons learned** (Ekirapa-Kiracho et al., 2014 ; Hawkes et al., 2016; Vogel & Punton, 2017)<br>• **#/type of written and/or other forms of knowledge exchange events** (newsletter, website summary, interim report, webinar, workshop, presentation, etc.) (ESSENCE on Health Research, 2016; Anita Kothari, MacLean, Edwards, & Hobbs, 2017; Ottoson et al., 2009; R. Makkar et al., 2016)<br>• #/type of innovative approaches of the WHO Secretariat to reach out for regular information sharing (Hanley et al., 2012; McLean & Tucker, 2013; The Networks of Centres of Excellence Secretariat, 2008)	• EVIPNet Europe Annual reports, WHO reporting, performance tracking sheet

* Indicators **in bold are required**, others are recommended. Indicators without reference were recommended by EE stakeholders and/or the authors.

OUTCOMES (short-term)	INDICATORS*	DATA COLLECTION METHODS
**Network membership growth and strengthening:** Network growth and stability	• **# of EVIPNet Europe member countries with succession planning** (Creech & Ramji, 2004)<br>• **# of network members who participated in network (training) activities** (Chapman, 2012; de-Graft Aikins et al., 2012; Peirson et al., 2013)<br>• **All members are clear about and share the purpose of the network and its role** (Sources4Network, 2016)<br>• **Individual knowledge and skills of network members to assess and critically analyse evidence** (Hawkes et al., 2016)<br>• #/types of requests from stakeholders for KT support and training from the country team/ KTP (Cole et al., 2016)	• EVIPNet Europe Annual reports, WHO reporting, country team/KTP annual activities and outputs inventory<br>• Qualitative interviews, online evaluation (survey)<br>• KTP annual activities and outputs inventory
**Internal and external visibility:** EVIPNet Europe seen as an expert in the KT field, adding value	• **# of invitations to conferences EVIPNet Europe (WHO Secretariat and country team/KTP) presented at** (Cole et al., 2016; ESSENCE on Health Research, 2016; Morton et al., 2012; The Networks of Centres of Excellence Secretariat, 2008)<br>• **#/type of publications by EVIPNet Europe** (including peer-reviewed publication and conference publications) (Angulo-Tuesta et al., 2016; ESSENCE on Health Research, 2016; Mahmood et al., 2011; Panel on Return on Investment in Health Research, 2009; The Networks of Centres of Excellence Secretariat, 2008; United Nations Development Programme)<br>• **# of citations of EVIPNet Europe authored publications by WHO Secretariat and country team/KTP**) (Angulo-Tuesta et al., 2016; Ekirapa-Kiracho et al., 2014 ; Global Development Network, 2017; Kwan et al., 2007; Mahmood et al., 2011; Panel on Return on Investment in Health Research, 2009)<br>• **Network was acknowledged internally by its members for its impact** (Ottoson et al., 2009)<br>• **Network was acknowledged by external stakeholders as useful for EIP** (Hanley et al., 2012; Murnaghan et al., 2013) **and catalysing change** (Ottoson et al., 2009; Sources4Network, 2016)<br>• # of case studies/success stories/good practice documents and/or lessons learnt documented and publicly shared (Bartlett & Peach, 2008; Hanley et	• EVIPNet Europe Annual reports, WHO reporting, performance tracking<br>• EVIPNet Europe Annual reports, WHO reporting, performance tracking, citation tracking<br>• Qualitative interviews, online evaluation (survey)<br>• EVIPNet Europe Annual reports, case studies<br>• EVIPNet Europe annual reports, WHO reporting, performance tracking, multicountry meetings, yammer

* Indicators **in bold are required**, others are recommended. Indicators without reference were recommended by EE stakeholders and/or the authors.

	al., 2012; Sources4Network, 2016; The Networks of Centres of Excellence Secretariat, 2008; Vogel & Punton, 2016, 2017)	
**Country teams/KTPs established and operationalized:** EVIPNet Europe established and KT work initiate	• **# of formal country teams/KTPs established** (Creech & Ramji, 2004; ESSENCE on Health Research, 2016; Hawkes et al., 2016; McLean & Tucker, 2013; The Networks of Centres of Excellence Secretariat, 2008; Vogel & Punton, 2017)<br>• **# of leaders and champions engaged to advocate for EIP** (Makkar et al., 2016; Sources4Network, 2016; Vogel & Punton, 2017)<br>• **# of network members who used resources/tools/publications of EVIPNet Europe** (Peirson et al., 2013; Waqa, Mavoa, Snowdon, Moodie, Schultz, et al., 2013)<br>• **Strategic plan** (ESSENCE on Health Research, 2016; Makkar et al., 2016), a**ctionable work plan** (Creech & Ramji, 2004)<br>• Compositions/representations of various sectors and disciplines to achieve the right mix of skills and abilities among members (Creech & Ramji, 2004; The Networks of Centres of Excellence Secretariat, 2008) and areas of work (The Networks of Centres of Excellence Secretariat, 2008)<br>• # of network members trained in KT management (ESSENCE on Health Research, 2016)<br>• #/types of requests from stakeholders for KT support and training (Cole et al., 2016)<br>• #/types of collaborators and their affiliations with country teams/KTPs (The Networks of Centres of Excellence Secretariat, 2008; Yazdizadeh, Majdzadeh, Alami, & Amrolalaei, 2014)<br>• **#/types of formal interactions between researchers and policy-makers (e.g. through journal clubs, roundtables, workshops, or focus groups etc)** (Hawkes et al., 2016; Johnson & Lavis, 2010; Makkar et al., 2016; R. Makkar et al., 2016)<br>• Routine reporting mechanism of M&E data through annual reports and lessons learnt(Ongolo-Zogo et al., 2014)	• EVIPNet Europe annual reports, WHO reporting, performance tracking, multicountry meetings, qualitative interviews, survey (online)<br>• Country teams/KTPs work plans<br>• EVIPNet Europe annual reports, WHO reporting, performance tracking, multicountry meetings, qualitative interviews, survey (online)<br>• Country team/KTP annual activities and outputs inventory

* Indicators **in bold are required**, others are recommended. Indicators without reference were recommended by EE stakeholders and/or the authors.

**Strengthened collaboration and partnerships:** EVIPNet Europe established collaborative projects, partnerships and networking opportunities, both network-internal and external	• **# of people accessing the virtual platform, # downloads & uploads; total # of times a partner/individual is mentioned by others** (Ekirapa-Kiracho et al., 2014 ; Global Development Network, 2017; Peirson et al., 2013)<br>• **Country-specific lessons learnt and M&E findings shared with network members** (Hanley et al., 2012)<br>• **Examples of one-off and ongoing peer support activities** (Global Development Network, 2017; Hanley et al., 2012; Sources4Network, 2016)<br>• **# of partnership policies** (joint documents such as terms of reference, joint action plans, rules around competition/collaboration, etc) **and/or MoU established between the WHO Secretariat and partners** (ESSENCE on Health Research, 2016; Anita Kothari et al., 2017; A.; Kothari, Sibbald, & Wathen, 2014)<br>• **# of joint publications or other activities by topic and country** (Alberta Addiction and Mental Health Research Partnership Program, 2014; Global Development Network, 2017; Hanley et al., 2012; Anita Kothari et al., 2017; Panel on Return on Investment in Health Research, 2009; The Networks of Centres of Excellence Secretariat, 2008; Yazdizadeh et al., 2014)	• Virtual platform analysis: Website tracking and social network analysis<br>• Country team/KTP annual activities and outputs inventory, case studies, yammer, WHO website<br>• EVIPNet Europe annual reports, WHO reporting, performance tracking, operational plans<br>• EVIPNet Europe annual reports, WHO reporting, performance tracking, WHO website

OUTCOMES (intermediate)	INDICATORS*	DATA COLLECTION METHODS
**Network member satisfaction:** Member countries satisfied with EVIPNet Europe and its activities	• **Opinions of program participants on the support given to member countries** (funds, agreement, advice and direction) (The Networks of Centres of Excellence Secretariat, 2008)<br>• Perception of EVIPNet Europe members of transparency/accountability in decision-making (Hanley et al., 2012)<br>• Perception of EVIPNet Europe members of equal treatment throughout the network (Hanley et al., 2012)<br>• **Level of needs, satisfaction and values reported by the country team/KTP and external stakeholders** (Anita Kothari et al., 2017; A.; Kothari et al., 2014; The CIPHER Investigators, 2014)	• Qualitative interviews, survey (online)
**Demonstration of efficiency and effectiveness in KT:** EVIPNet Europe showed to be efficient and effective through its KT activities	• **Achievement of objectives by EVIPNet Europe (qualitative estimate, %)** (Global Development Network, 2017)<br>• % of WHO Secretariat budget spent on management, administration, staff support (Hanley et al., 2012)	• EVIPNet Europe annual reports, WHO reporting, performance tracking, operational plans, qualitative interviews

* Indicators **in bold are required**, others are recommended. Indicators without reference were recommended by EE stakeholders and/or the authors.

	• % of administrative costs of total costs in comparison to other networking programs (The Networks of Centres of Excellence Secretariat, 2008)<br>• **Budget structure of the WHO Secretariat aligned with the work priorities and income streams** (The Joanna Briggs Institute, 2015)<br>• # of successful funding applications (de-Graft Aikins et al., 2012; ESSENCE on Health Research, 2016; Ongolo-Zogo et al., 2014; Ottoson et al., 2009; Panel on Return on Investment in Health Research, 2009; Sallis et al., 2009)	• EVIPNet Europe financial statements, economic evaluation (value for money analysis)<br>• Website analytics
**Institutionalization of sustainable KTPs:** National and regional networks becoming self-sustaining	• **Plans are in place for succession of country teams/KTP leadership** (Creech & Ramji, 2004) and/or retention (R. Makkar et al., 2016)<br>• KTPs viewed their work as a long-term initiative (El-Jardali, Lavis, Moat, Pantoja, & Ataya, 2014)<br>• Availability of adequate human resources (skills, skill-building opportunities, KT stakeholder representation) (ESSENCE on Health Research, 2016; Ottoson et al., 2009; R. Makkar et al., 2016; The Networks of Centres of Excellence Secretariat, 2008; Waqa, Mavoa, Snowdon, Moodie, Nadakuitavuki, et al., 2013)<br>• Availability of adequate financial resources (sustainable funding, financial management) (Cole et al., 2016; Creech & Ramji, 2004; ESSENCE on Health Research, 2016; Ottoson et al., 2009; R. Makkar et al., 2016; The Networks of Centres of Excellence Secretariat, 2008)	• EVIPNet Europe annual reports, WHO reporting, performance tracking, operational plans, qualitative interviews, survey (online)<br>• Qualitative interviews, survey (online)

(III) KT and EIP value and culture

OUTPUTS	INDICATORS*	DATA COLLECTION METHODS
**Production and dissemination of EIP action plan and promotion of KT values:** Publication of an action plan to strengthen the use of evidence, information and research for policy-making in the WHO European Region	• **Regional EIP Action Plan by WHO Secretariat adopted by Member States**<br>• **#/type of activity to promote the EIP Action Plan and its KT values** (presented at internal/external meetings and conferences, etc)	• EIP Action Plan<br>• EVIPNet Europe Annual reports, WHO reporting, performance tracking

* Indicators **in bold are required**, others are recommended. Indicators without reference were recommended by EE stakeholders and/or the authors.

OUTCOMES (short-term)	INDICATORS*	DATA COLLECTION METHODS
**Increased commitment to KT:** Heightened interest, growth of KT field and climate	• **Increased awareness of the importance of KT among policy-makers, stakeholders and researchers** (Cole et al., 2016; Conklin & Stolee, 2008; El-Jardali et al., 2014), **commitment and/or demand for KT capacity support** (Vogel & Punton, 2017)<br>• **EVIPNet Europe increasingly integrated into or working jointly with other regional programmes and initiatives** (WHO Regional Office for Europe, 2016)<br>• **European KT identity emerged** (partners get a better overview of what characterizes the European KT map and link up to it (European Implementation Collaborative, n. d.)<br>• **Stakeholders understand the impact the network is having, and actively promote this** (Sources4Network, 2016; Vogel & Punton, 2016)<br>• Policy-makers and influencers reported increased KT capacity, knowledge and skills (El-Jardali et al., 2014; ESSENCE on Health Research, 2016; Global Development Network, 2017; Hanley et al., 2012; The CIPHER Investigators, 2014; Vogel & Punton, 2016)<br>• # of emerging and/or increasing funding streams for the support of KT research and practice (Cole et al., 2016; ESSENCE on Health Research, 2016; European Implementation Collaborative, n. d.; Mahmood et al., 2011; Panel on Return on Investment in Health Research, 2009)<br>• # of new KT methodologies and approaches (European Implementation Collaborative, n. d.)<br>• # of KT and EIP curricula, masters and PhD programs in EVIPNet member countries (ESSENCE on Health Research, 2016; European Implementation Collaborative, n. d.; Panel on Return on Investment in Health Research, 2009; Vogel & Punton, 2016)	• Qualitative interviews, survey (online)<br>• Mapping of regional programmes and initiatives<br>• Qualitative interviews, survey (online)<br>• Mapping of environment

* Indicators **in bold are required**, others are recommended. Indicators without reference were recommended by EE stakeholders and/or the authors.

OUTCOMES (intermediate)	INDICATORS*	DATA COLLECTION METHODS
**Increased evidence use by society and KT values:** Evidence use increasingly mainstreamed through society	• **Increased # of KT processes across organizational, regional, national boundaries (e.g. conferences, seminars, projects)** (European Implementation Collaborative, n. d.)<br>• # of citations in advocacy publications: Research mentioned in publications (leaflets etc.) produced by advocacy groups, including patient organizations (Panel on Return on Investment in Health Research, 2009)<br>• # of citations by media: Continued media coverage of EVIPNet Europe and its activities (e.g. # of media mentions, website visits) (Alberta Addiction and Mental Health Research Partnership Program, 2014; Galluzzo et al., 2012; Global Development Network, 2017; Panel on Return on Investment in Health Research, 2009; Vogel & Punton, 2016)	• Qualitative interviews, survey (online), country team/KTP annual activities and outputs inventory<br><br>• Citation tracking<br><br>• Media analysis

Abbreviations used in Appendix B

EBP - evidence brief for policy

EIP – evidence-informed policy-making

EVIPNet – Evidence-informed Policy Network

IRWFY - *Is Research Working for You?*

KT – knowledge translation

KTP – knowledge translation platform

M&E – monitoring and evaluation

ORACLe - *Organizational Research Access, Culture and Leadership*

PD – policy dialogue

PhD – Doctor of Philosophy

SA – situation analysis

WHO – World Health Organization

* Indicators **in bold are required**, others are recommended. Indicators without reference were recommended by EE stakeholders and/or the authors.

References

Alberta Addiction and Mental Health Research Partnership Program. (2014). *Knowledge Translation Evaluation Planning Guide*. Edmonton, AB: Alberta Health Services.

Angulo-Tuesta, A., Santos, L. M., & Natalizi, D. A. (2016). Impact of health research on advances in knowledge, research capacity-building and evidence-informed policies: a case study on maternal mortality and morbidity in Brazil. *Sao Paulo Med J, 134*(2), 153-162. doi:10.1590/1516-3180.2015.01530211

Araujo de Carvalho, I., Byles, J., Aquah, C., Amofah, G., Biritwum, R., Panisset, U., . . . Beard, J. (2015). Informing evidence-based policies for ageing and health in Ghana. *Bull World Health Organ, 93*(1), 47-51. doi:10.2471/BLT.14.136242

Bartlett, H., & Peach, L. C. (2008). 'I went in feeling like a student and came out feeling like a researcher'. An evaluation of the first Australian Masterclass for Emerging Researchers in Ageing. *Australas J Ageing, 27*(4), 195-199. doi:10.1111/j.1741-6612.2008.00318.x

Chapman, E. (2012). *Evaluation of the Evidence Informed Policy Networks (EVIPNet)*. Washington, D.C.: PAHO.

Cole, D. C., Nyirenda, L. J., Fazal, N., & Bates, I. (2016). Implementing a national health research for development platform in a low-income country - a review of Malawi's Health Research Capacity Strengthening Initiative. *Health Res Policy Syst, 14*, 24. doi:10.1186/s12961-016-0094-3

Conklin, J., & Stolee, P. (2008). Un modèle d'évaluation du partage de connaissances en contexte de réseau. *CJNR, 40*(2), 116–124.

Creech, H., & Ramji, A. (2004). *Knowledge Networks: Guidelines for Assessment*. Winnipeg, Manitoba: International Institute for Sustainable Development.

de-Graft Aikins, A., Arhinful, D. K., Pitchforth, E., Ogedegbe, G., Allotey, P., & Agyemang, C. (2012). Establishing and sustaining research partnerships in Africa: a case study of the UK-Africa Academic Partnership on Chronic Disease. *Global Health, 8*, 29. doi:10.1186/1744-8603-8-29

Ekirapa-Kiracho, E., Walugembe, D., Tetui, M., Kisakye, A., Rutebemberwa, E., Sengooba, F., . . . Kiwanuka, S. N. (2014). Evaluation of a health systems knowledge translation network for Africa (KTNET): a study protocol. *Implement Sci, 9*(170).

El-Jardali, F., Lavis, J. N., Moat, K., Pantoja, T., & Ataya, N. (2014). Capturing lessons learned from evidence-to-policy initiatives through structured reflection. *Heal Res Policy Syst, 12*(2).

ESSENCE on Health Research. (2016). *Planning, Monitoring and Evaluation Framework for Research Capacity Strengthening*. Geneva: Training in Tropical Diseases (TDR)/World Health Organization (WHO).

European Implementation Collaborative. (n. d.). EIC Logic Model. In: European Implementation Collaborative.

Galluzzo, L., Scafato, E., Martire, S., Anderson, P., Colom, J., Segura, L., . . . Group, f. t. V. p. W. (2012). Alcohol and older people. The European project vintage: Good Health Into Older Age. *Ann Ist Super Sanita, 48*(48), 221–231.

Global Development Network. (2017). The Road Ahead. Strategy 2017-2022. Local Research for Better Lives. In. Washington, D.C.: GDN.

Hanley, T., Gould, C., Harle, J., & Nelson, K. (2012). *International Network for the Availability of Scientific Publications. Programme for the Enhancement of Research Information. Phase II. External Evaluation 2008-12. Final Report.* Oxford: International Network for the Availability of Scientific Publications.

Hawkes, S., B., K. A., Jadeja, N., Jimenez, M., Buse, K., Anwar, I., . . . Whitworth, J. (2016). Strengthening capacity to apply health research evidence in policy making: experience from four countries. *Health Policy Plan, 31*(2), 161-170. doi:10.1093/heapol/czv032

Johnson, N. A., & Lavis, J. N. (2010). *Outcomes Evaluation. Procedures Manual for for Evaluating Knowledge-Translation Platforms in Low- and Middle- Income Countries.* (M. U. P. i. P. Decision-Making Ed.). Hamilton, Canada: McMaster University Program in Policy Decision-Making.

Kothari, A., MacLean, L., Edwards, N., & Hobbs, A. (2017). Indicators at the interface: managing policymaker-researcher collaboration. *Knowledge Management Research & Practice, 9*(3), 203-214. doi:10.1057/kmrp.2011.16

Kothari, A., Sibbald, S., & Wathen, C. N. (2014). Evaluation of partnerships in a transnational family violence prevention network using an integrated knowledge translation and exchange model: a mixed methods study. *Heal Res Policy Syst, 12*(25).

Kwan, P., Johnston, J., Fung, A. Y., Chong, D. S., Collins, R. A., & Lo, S. V. (2007). A systematic evaluation of payback of publicly funded health and health services research in Hong Kong. *BMC Health Serv Res, 7*, 121. doi:10.1186/1472-6963-7-121

Mahmood, S., Hort, K., Ahmed, S., Salam, M., & Cravioto, A. (2011). Strategies for capacity building for health research in Bangladesh: Role of core funding and a common monitoring and evaluation framework. *Health Res Policy Syst, 9*(31).

Makkar, S. R., Turner, T., Williamson, A., Louviere, J., Redman, S., Haynes, A., . . . Brennan, S. (2016). The development of ORACLe: a measure of an organisation's capacity to engage in evidence-informed health policy. *Health Res Policy Syst, 14*, 4. doi:10.1186/s12961-015-0069-9

Mavoa, H., Waqa, G., Moodie, M., Kremer, P., McCabe, M., Snowdon, W., & Swinburn, B. (2012). Knowledge exchange in the Pacific: The TROPIC (Translational Research into Obesity Prevention Policies for Communities) project. *BMC Public Health, 12*, 552. doi:10.1186/1471-2458-12-552

McLean, R., & Tucker, J. (2013). *Evaluation of CIHR's Knowledge Translation Funding Program*. Ottawa: Canadian Institutes of Health Research.

Morton, J., Shaxson, L., & Greenland, J. (2012). *Final Report. Process Evaluation of the International Initiative for Impact Evaluation (2008-11)*. London: Triple Line Consulting Ltd/Overseas Development Institute.

Murnaghan, D., Morrison, W., Griffith, E. J., Bell, B. L., Duffley, L. A., McGarry, K., & Manske, S. (2013). Knowledge exchange systems for youth health and chronic disease prevention: a tri-provincial case study. *Chronic Diseases and Injuries in Canada, 33*(4), 257-266.

Neves, J., Lavis, J. N., Panisset, U., & Klint, M. H. (2014). Evaluation of the international forum on evidence informed health policymaking: Addis Ababa, Ethiopia – 27 to 31 August 2012. *Health Res Policy Syst, 12*(14).

Ongolo-Zogo, P., Lavis, J. N., Tomson, G., & Sewankambo, N. K. (2014). Initiatives supporting evidence informed health system policymaking in Cameroon and Uganda: a comparative historical case study. *BMC Health Services Research, 14*(612).

Ottoson, J. M., Green, L. W., Beery, W. L., Senter, S. K., Cahill, C. L., Pearson, D. C., . . . Leviton, L. (2009). Policy-contribution assessment and field-building analysis of the Robert Wood Johnson Foundation's Active Living Research Program. *Am J Prev Med, 36*(2 Suppl), S34-43. doi:10.1016/j.amepre.2008.10.010

Panel on Return on Investment in Health Research. (2009). *Making an Impact. A Preferred Framework and Indicators to Measure Returns on Investment in Health Research.* (C. A. o. H. Sciences Ed.). Ottawa, ON: Canadian Academy of Health Sciences.

Peirson, L., Catallo, C., & Chera, S. (2013). The Registry of Knowledge Translation Methods and Tools: a resource to support evidence-informed public health. *Int J Public Health, 58*(4), 493-500. doi:10.1007/s00038-013-0448-3

R. Makkar, S., Brennan, S., Turner, T., Williamson, A., Redman, S., & Green, S. (2016). The development of SAGE: A tool to evaluate how policymakers' engage with and use research in health policymaking. *Research Evaluation, 25*(3), 315-328. doi:10.1093/reseval/rvv044

Sallis, J. F., Linton, L. S., Kraft, M. K., Cutter, C. L., Kerr, J., Weitzel, J., . . . Pratt, M. (2009). The Active Living Research program: six years of grantmaking. *Am J Prev Med, 36*(2 Suppl), S10-21. doi:10.1016/j.amepre.2008.10.007

Sources4Network. (2016). Network Maturity Matrix. In. London: NHS England [website]. https://www.source4networks.org.uk/images/site/files/Maturity_Model_Matrix_v2_071216-FINAL.pdf.

The CIPHER Investigators. (2014). Supporting Policy In health with Research: an Intervention Trial (SPIRIT)-protocol for a stepped wedge trial. *BMJ Open, 4*(7), e005293. doi:10.1136/bmjopen-2014-005293

The Joanna Briggs Institute. (2015). *2016 - 2020 Strategic Plan. Better evidence. Better outcomes. Brighter future. Taking JBI to 2020 and beyond…* Adelaide: The University of Adelaide.

The Networks of Centres of Excellence Secretariat. (2008). *Joint Results-based Management and Accountability Framework and Risk-Based Audit Framework for the Class Grant Networks of Centres of Excellence Program*. Ottawa: The Networks of Centres of Excellence Secretariat.

Uneke, C. J., Ezeoha, A. E., Ndukwe, C. D., Oyibo, P. G., & Onwe, F. (2011). Enhancing health policymakers' capacity to use information and communication technology in Nigeria. *Health Inform Dev Ctries, 5*(2), 228–246.

Uneke, C. J., Ezeoha, A. E., Uro-Chukwu, H., Ezeonu, C. T., Ogbu, O., Onwe, F., & Edoga, C. (2015a). Enhancing the Capacity of Policy-Makers to Develop Evidence-Informed Policy Brief on Infectious Diseases of Poverty in Nigeria. *Int J Health Policy Manag, 4*(9), 599-610. doi:10.15171/ijhpm.2015.100

Uneke, C. J., Ezeoha, A. E., Uro-Chukwu, H., Ezeonu, C. T., Ogbu, O., Onwe, F., & Edoga, C. (2015b). Improving Nigerian health policymakers' capacity to access and utilize policy relevant evidence: outcome of information and communication technology training workshop. *Pan Afr Med J, 21*, 212. doi:10.11604/pamj.2015.21.212.6375

United Nations Development Programme. (1997). *Capacity Development. Management Development and Governance Division.* New York: United Nations Development Programme.

Vogel, I., & Punton, M. (2016). *Building Capacity to Use Ressearch Evidence (BCURE) Evaluation: Stage 1 Synthesis Report*. Brighton: Itad.

Vogel, I., & Punton, M. (2017). *Building Capacity to Use Research Evaluation (BCURE) realist evaluation: Stage 2 Synthesis Report*: ITAD.

Waqa, G., Mavoa, H., Snowdon, W., Moodie, M., Nadakuitavuki, R., Mc Cabe, M., & Swinburn, B. (2013). Participants' perceptions of a knowledge-brokering strategy to facilitate evidence-informed policy-making in Fiji. *BMC Public Health, 13*(725).

Waqa, G., Mavoa, H., Snowdon, W., Moodie, M., Schultz, J., McCabe, M., . . . Swinburn, B. (2013). Knowledge brokering between researchers and policymakers in Fiji to develop policies to reduce obesity: A process evaluation. *Implement Sci, 8*(74).

WHO Regional Office for Europe. (2016). *Action plan to strengthen the use of evidence, information and research for policy-making in the WHO European Region. (EUR/RC66/12)*. Copenhagen: WHO Regional Office for Europe

Yazdizadeh, B., Majdzadeh, R., Alami, A., & Amrolalaei, S. (2014). How can we establish more successful knowledge networks in developing countries? Lessons learnt from knowledge networks in Iran. *Heal Res Policy Syst, 12*(63).
