# Supplementary material for: A comprehensive monitoring and evaluation framework for evidence to policy networks
Source: Eval Program Plann. Author manuscript; Available in PMC 2023 Jan 12. (PMC7614046; doi:10.1016/j.evalprogplan.2022.102053)
Supplement: Appendix_C_Manuscript KT framework_KTP indicators [file EMS159051-supplement-Appendix_C_Manuscript_KT_framework_KTP_indicators.pdf]

Appendix C. EVIPNet Europe M&E framework: Country team/KTP indicators

I) KT capacity and skill building

OUTPUTS	INDICATORS*	DATA COLLECTION METHODS
**Capacity building for country teams/Knowledge Translation Platforms (KTP) and external stakeholders** convened by the Evidence-informed Policy Network (EVIPNet) Europe country teams/KTPs: 1) Skill-building workshops on knowledge translation (KT) (search/access/appraise/synthesize/use evidence) and training facilitation skills attended by the country team/ KTP and 2) KT skills building workshops for external national stakeholders organized by the country team/ KTP	• **#/types of WHO KT skill-building workshops attended by the country team/KTP** (Chapman, 2012; Ongolo-Zogo, Lavis, Tomson, & Sewankambo, 2014; Sallis et al., 2009; Waqa, Mavoa, Snowdon, Moodie, Schultz, et al., 2013)<br>• **#/types of country team/KTP participants** (Chapman, 2012; de-Graft Aikins et al., 2012; Neves, Lavis, Panisset, & Klint, 2014; Ongolo-Zogo et al., 2014; C.J.; Uneke, Ezeoha, Ndukwe, Oyibo, & Onwe, 2011; C. J. Uneke et al., 2015a, 2015b; Waqa, Mavoa, Snowdon, Moodie, Nadakuitavuki, et al., 2013; Waqa, Mavoa, Snowdon, Moodie, Schultz, et al., 2013), by gender (C.J.; Uneke et al., 2011; C. J. Uneke et al., 2015a, 2015b; Waqa, Mavoa, Snowdon, Moodie, Schultz, et al., 2013)<br>• **#/types of KT workshops organized by country team/KTP members** (Ongolo-Zogo et al., 2014; Sallis et al., 2009; The CIPHER Investigators, 2014)<br>• **#/types of external stakeholder participants** (Bartlett & Peach, 2008; de-Graft Aikins et al., 2012; ESSENCE on Health Research, 2016; Neves et al., 2014; Ongolo-Zogo et al., 2014; C.J.; Uneke et al., 2011; C. J. Uneke et al., 2015a, 2015b; Waqa, Mavoa, Snowdon, Moodie, Nadakuitavuki, et al., 2013), by gender (ESSENCE on Health Research, 2016; C.J.; Uneke et al., 2011; C. J. Uneke et al., 2015a, 2015b; Waqa, Mavoa, Snowdon, Moodie, Schultz, et al., 2013)<br>• **Participants' satisfaction scores** (Bartlett & Peach, 2008; Neves et al., 2014; C.J.; Uneke et al., 2011; C. J. Uneke et al., 2015a, 2015b; Waqa, Mavoa, Snowdon, Moodie, Nadakuitavuki, et al., 2013)<br>• #/types of events/meetings attended by external stakeholders (Ongolo-Zogo et al., 2014)	• Evidence-informed Policy Network (EVIPNet) Europe annual reports, performance tracking sheet, workshop evaluation report<br>• Workshop evaluation reports, training feedback forms (initial and 6-12 month follow up), qualitative interviews
**KT products and activities developed by country/teams/KTP:** Health policy topic issues prioritized; evidence briefs for policy (EBPs) on priority health issues developed; policy dialogues organized	• **Types/topics of health policy priorities for evidence briefs for policy (EBPs)** (Araujo de Carvalho et al., 2015; Johnson & Lavis, 2010; Ongolo-Zogo et al., 2014)<br>• **#/% of peer reviewed EBPs published by KTP** (Chapman, 2012; Ekirapa-Kiracho et al., 2014 ; El-Jardali, Lavis, Moat, Pantoja, & Ataya, 2014)<br>• **# of policy dialogues held by KTP/country team** (Ekirapa-Kiracho et al., 2014 ; El-Jardali et al., 2014; Ongolo-Zogo et al., 2014; R. Makkar et al., 2016)	• EVIPNet Europe annual report; country team/KTP annual activities and outputs inventory

* Indicators **in bold are required**, others are recommended. Indicators without reference were recommended by EE stakeholders and/or the authors.

	• **# of KT products co-produced by KTP with policy-makers** (The Networks of Centres of Excellence Secretariat, 2008)	
**Monitoring and evaluation (M&E) plan:** Mechanism for regular M&E in place	• **Clearly defined processes and systems to conduct and/or commission monitoring and evaluations** (Makkar et al., 2016; R. Makkar et al., 2016)	• Country team/KTP M&E plan developed

OUTCOMES (short-term)	INDICATORS*	DATA COLLECTION METHODS
**Increased KT capacity:** Increased KT knowledge and skills of country teams/Knowledge Translation Platforms (KTP) and external stakeholders to search/access/appraise/synthesize/use research evidence	• **% of workshop participants' feedback on:**<br>**- change in knowledge** (Neves et al., 2014; Sallis et al., 2009; C.J.; Uneke et al., 2011; C. J. Uneke et al., 2015a, 2015b)<br>**- change in skills** (Neves et al., 2014; Ottoson et al., 2009; C.J.; Uneke et al., 2011; C. J. Uneke et al., 2015a, 2015b)<br>• **Policy-makers and influencers reported increased KT capacity, knowledge and skills** (El-Jardali et al., 2014; The CIPHER Investigators, 2014)	• Workshop evaluation reports, training feedback forms (initial and 6-12 month follow up), qualitative interviews<br><br>• Qualitative interviews, survey (online)
**Increased production and visibility of KT products** produced by KTP, searchable and discoverable	• **Change in # of KT products produced (according to type)**<br>• **Change in # of KT products published (according to type)**<br>• Relevance to (Creech & Ramji, 2004; Ekirapa-Kiracho et al., 2014 ; R. Makkar et al., 2016) and timeliness (Creech & Ramji, 2004) of EBPs in priority health issues<br>**•#/types of publications and conferences attended by the country team/KTP members** (Angulo-Tuesta, Santos, & Natalizi, 2016; Cole, Nyirenda, Fazal, & Bates, 2016; Ekirapa-Kiracho et al., 2014 ; Kwan et al., 2007; Mahmood, Hort, Ahmed, Salam, & Cravioto, 2011; Sallis et al., 2009; Waqa, Mavoa, Snowdon, Moodie, Schultz, et al., 2013)	• Country team/KTP annual activities and outputs inventory, WHO website tracking<br>• Qualitative interviews, survey (online)<br><br>• Country team/KTP annual activities and outputs inventory, WHO website tracking
**Routine M&E** by the country team/ KTP	• # of times the country team/KTP measured perceived evidence-informed policy (EIP) skills/changes in skills (using IRWFY) (Mavoa et al., 2012; Waqa, Mavoa, Snowdon, Moodie, Schultz, et al., 2013)<br>• # of times the country team/KTP assessed capacity (using ORACLe, Organizational Research Access, Culture and Leadership) (Makkar et al., 2016; The CIPHER Investigators, 2014)	• IRWFY findings<br><br>• ORACLe findings

OUTCOMES (intermediate)	INDICATORS*	DATA COLLECTION METHODS

* Indicators **in bold are required**, others are recommended. Indicators without reference were recommended by EE stakeholders and/or the authors.

**Increased use of KT products** at national and Regional levels	• **# of citations of KT products (in policy, research and/or media documents in speeches, debates and informal internal documents)** (Angulo-Tuesta et al., 2016; Mahmood et al., 2011)<br>• **Reported use** (e.g. evidence presented and/or discussed in meetings, included as a technical document in policy formulation meeting, referenced in speeches, debates and informal internal documents, etc.) (Araujo de Carvalho et al., 2015; Ekirapa-Kiracho et al., 2014 ; Mahmood et al., 2011; Murnaghan et al., 2013; Ottoson et al., 2009; The CIPHER Investigators, 2014)	• Website tracking and social network analysis<br><br>• Qualitative interviews, EVIPNet Europe Annual reports, Online evaluation (survey), success stories
**Improved country team/KTP performance:** Lessons learnt and good practice used, applied and shared by KTP for improving KTP and overall network operationalization	• **# of case studies/good practice documents and/or lessons learnt documented and publicly shared** (Bartlett & Peach, 2008; Sallis et al., 2009)	• Country team/KTP annual activities and outputs inventory

(II) Network (structure, governance, and leadership)

OUTPUTS	INDICATORS*	DATA COLLECTION METHODS
**Country team/KTP engaged with EVIPNet Europe:** Situation analysis (SA) conducted, stakeholder meetings held	• **SA conducted by the country team/KTP** (Araujo de Carvalho et al., 2015)	• Country team/KTP annual activities and outputs inventory
**Country team/KTP structure developed:** Resources, Governance structure put in place, management and leadership, A formal/informal country team/ KTP is established	• **Availability of a memorandum of understanding (MoU) or equivalent for a formal country team/KTP** (ESSENCE on Health Research)<br>• **Credible KT champions with dedicated time available** (Sources4Network) with a clear vision for EIP (Makkar et al., 2016)<br>• **Compositions/representations of various sectors and disciplines to achieve the right mix of skills and abilities among members** (Creech & Ramji, 2004), areas of work (The Networks of Centres of Excellence Secretariat, 2008) and	• MoU<br><br><br><br>• Country Team/KTP Profile

* Indicators **in bold are required**, others are recommended. Indicators without reference were recommended by EE stakeholders and/or the authors.

	gender (ESSENCE on Health Research, 2016; C.J.; Uneke et al., 2011; C. J. Uneke et al., 2015a, 2015b; Waqa, Mavoa, Snowdon, Moodie, Schultz, et al., 2013)<br>• # of network members trained in KT management (ESSENCE on Health Research, 2016)	• Country team/KTP annual activities and outputs inventory
**Mechanisms for collaboration and peer-exchange** established	• **#/types of visits and posts on Yammer by country team/KTP members** (de-Graft Aikins et al., 2012)<br>• **# and type of network-wide events the country team/KTP participated in** (de-Graft Aikins et al., 2012; ESSENCE on Health Research, 2016; Waqa, Mavoa, Snowdon, Moodie, Schultz, et al., 2013)<br>• **# and type of collaborations initiated between the country team/KTP and national/international KT institutions** (de-Graft Aikins et al., 2012; Ekirapa-Kiracho et al., 2014 ; ESSENCE on Health Research, 2016; Ottoson et al., 2009)<br>• **#/types of formal interactions between researchers and policy-makers (e.g. through journal clubs, roundtables, workshops, or focus groups etc)** (Hawkes et al., 2016; Johnson & Lavis, 2010; Makkar et al., 2016; R. Makkar et al., 2016)	• Website tracking and social network analysis<br>• Country team/KTP annual activities and outputs inventory, WHO reporting<br>• Country team/KTP annual activities and outputs inventory

OUTCOMES (short-term)	INDICATORS*	DATA COLLECTION METHODS
**Operationalization of KTP**	• **Country teams/KTP members share the same ambition and vision for the network** (Sources4Network, 2016)<br>• **Strategic plan (ESSENCE on Health Research, 2016; Makkar et al., 2016), actionable work plan** (Creech & Ramji, 2004)<br>• #/types of requests from stakeholders for KT support and training (Cole et al., 2016) and evidence (Ekirapa-Kiracho et al., 2014 ; Garforth, Ozor, Usher, & Bell, 2014; Langlois et al., 2016; Waqa, Mavoa, Snowdon, Moodie, Schultz, et al., 2013)<br>• # of network members who used resources/tools/publications of EVIPNet Europe (Chapman, 2012; Peirson, Catallo, & Chera, 2013; Waqa, Mavoa, Snowdon, Moodie, Schultz, et al., 2013)<br>• Routine reporting mechanism of M&E data through annual reports and lessons learnt (Ongolo-Zogo et al., 2014)	• Qualitative interviews<br>• Country team/KTP work plan<br>• Country team/KTP annual activities and outputs inventory

* Indicators **in bold are required**, others are recommended. Indicators without reference were recommended by EE stakeholders and/or the authors.

**Production of collaborative activities and stakeholder engagement** by the country team/KTP	• **# of joint country team/KTP publications or other activities by topic** (ESSENCE on Health Research, 2016; Yazdizadeh, Majdzadeh, Alami, & Amrolalaei, 2014)<br>• **# of one-off and ongoing peer support activities** (Sources4Network, 2016)<br>• **Country-specific lessons learnt and M&E findings shared with network members** (Hanley, Gould, Harle, & Nelson, 2012)<br>• **#/types of formal interactions with researchers** (e.g. through journal clubs, roundtables, workshops, or focus groups etc) (Hawkes et al., 2016; Johnson & Lavis, 2010; Makkar et al., 2016)	• Country team/KTP annual activities and outputs inventory

OUTCOMES (intermediate)	INDICATORS*	DATA COLLECTION METHODS
**Institutionalization of sustainable KTP:** Regional and national networks are self-sustainable and autonomous	• **Plan is in place for succession of country teams/KTP leadership** (Creech & Ramji, 2004)<br>• **Availability of adequate human resources** (skills, skill-building opportunities, KT stakeholder representation) (Ekirapa-Kiracho et al., 2014 ; Ottoson et al., 2009; Waqa, Mavoa, Snowdon, Moodie, Nadakuitavuki, et al., 2013)<br>• **Availability of adequate financial resources** (sustainable funding, financial management) (Cole et al., 2016; Ottoson et al., 2009)	• Country teams/KTP succession plan<br>• Country team/KTP annual activities and outputs inventory<br>• Country team/KTP HR documents<br>• Country team/KTP HR budgetary documents

(III) KT and EIP value and culture

OUTPUTS	INDICATORS*	DATA COLLECTION METHODS
**Promotion of EIP action plan and KT values:** KT agenda setting and promotion through the EIP action plan	**#/types of events, meetings, conferences in which the action plan was discussed by the country team/KTPs at national level**	• Country team/KTP annual activities and outputs inventory

* Indicators **in bold are required**, others are recommended. Indicators without reference were recommended by EE stakeholders and/or the authors.

OUTCOMES (short-term)	INDICATORS*	DATA COLLECTION METHODS
**Increased commitment to KT:** Heightened interest, growth of KT field and climate	• **Awareness of the importance of KT among national policy-makers, stakeholders and researchers** (Cole et al., 2016; Conklin & Stolee, 2008; El-Jardali et al., 2014; Ottoson et al., 2009) **and demand for KT capacity support** (Vogel & Punton, 2017)<br>• **National stakeholders understand the impact the network is having, and actively promote this** (Sources4Network, 2016)<br>• **National policy-makers and influencers report increased KT capacity, knowledge and skills** (El-Jardali et al., 2014; Langlois et al., 2016)<br>• # KT and EIP university curricula developed (Vogel & Punton, 2016)<br>• # of emerging funding streams for the support of KT research and practice (Cole et al., 2016; ESSENCE on Health Research, 2016)	• Qualitative interviews, survey (online)<br>• Mapping of environment

OUTCOMES (intermediate)	INDICATORS*	DATA COLLECTION METHODS
**Increased evidence use by society and KT values:** Evidence use increasingly mainstreamed through society	• **Indications of behaviour change of national researchers and knowledge-users in response to the work of the country team/KTP** (Conklin & Stolee, 2008)<br>• # of citations in advocacy publications: Research mentioned in national publications (leaflets etc.) produced by advocacy groups, including patient organizations (media citation analysis and citation in public policy documents) (Panel on Return on Investment in Health Research, 2009)<br>• Continued media coverage and promotion of the importance of evidence use and integration of KT (e.g. # of media mentions, website visits) (Bartlett & Peach, 2008; Galluzzo et al., 2012)	• Qualitative interviews, survey (online)<br>• Citation tracking<br>• Media analysis

* Indicators **in bold are required**, others are recommended. Indicators without reference were recommended by EE stakeholders and/or the authors.

Abbreviations used in Appendix C

EBP - evidence brief for policy

EIP – evidence-informed policy-making

EVIPNet– Evidence-informed Policy Network

IRWFY - *Is Research Working for You?*

KT – knowledge translation

KTP – knowledge translation platform

M&E – monitoring and evaluation

ORACLe - *Organizational Research Access, Culture and Leadership*

SA – situation analysis

WHO – World Health Organization

* Indicators **in bold are required**, others are recommended. Indicators without reference were recommended by EE stakeholders and/or the authors.

References

Angulo-Tuesta, A., Santos, L. M., & Natalizi, D. A. (2016). Impact of health research on advances in knowledge, research capacity-building and evidence-informed policies: a case study on maternal mortality and morbidity in Brazil. *Sao Paulo Med J, 134*(2), 153-162. doi:10.1590/1516-3180.2015.01530211

Araujo de Carvalho, I., Byles, J., Aquah, C., Amofah, G., Biritwum, R., Panisset, U., . . . Beard, J. (2015). Informing evidence-based policies for ageing and health in Ghana. *Bull World Health Organ, 93*(1), 47-51. doi:10.2471/BLT.14.136242

Bartlett, H., & Peach, L. C. (2008). 'I went in feeling like a student and came out feeling like a researcher'. An evaluation of the first Australian Masterclass for Emerging Researchers in Ageing. *Australas J Ageing, 27*(4), 195-199. doi:10.1111/j.1741-6612.2008.00318.x

Chapman, E. (2012). *Evaluation of the Evidence Informed Policy Networks (EVIPNet)*. Washington, D.C.: PAHO.

Cole, D. C., Nyirenda, L. J., Fazal, N., & Bates, I. (2016). Implementing a national health research for development platform in a low-income country - a review of Malawi's Health Research Capacity Strengthening Initiative. *Health Res Policy Syst, 14*, 24. doi:10.1186/s12961-016-0094-3

Conklin, J., & Stolee, P. (2008). Un modèle d'évaluation du partage de connaissances en contexte de réseau. *CJNR, 40*(2), 116–124.

Creech, H., & Ramji, A. (2004). *Knowledge Networks: Guidelines for Assessment*. Winnipeg, Manitoba: International Institute for Sustainable Development.

de-Graft Aikins, A., Arhinful, D. K., Pitchforth, E., Ogedegbe, G., Allotey, P., & Agyemang, C. (2012). Establishing and sustaining research partnerships in Africa: a case study of the UK-Africa Academic Partnership on Chronic Disease. *Global Health, 8*, 29. doi:10.1186/1744-8603-8-29

Ekirapa-Kiracho, E., Walugembe, D., Tetui, M., Kisakye, A., Rutebemberwa, E., Sengooba, F., . . . Kiwanuka, S. N. (2014). Evaluation of a health systems knowledge translation network for Africa (KTNET): a study protocol. *Implement Sci, 9*(170).

El-Jardali, F., Lavis, J. N., Moat, K., Pantoja, T., & Ataya, N. (2014). Capturing lessons learned from evidence-to-policy initiatives through structured reflection. *Heal Res Policy Syst, 12*(2).

ESSENCE on Health Research. (2016). *Planning, Monitoring and Evaluation Framework for Research Capacity Strengthening*. Geneva: Training in Tropical Diseases (TDR)/World Health Organization (WHO).

Galluzzo, L., Scafato, E., Martire, S., Anderson, P., Colom, J., Segura, L., . . . Group, f. t. V. p. W. (2012). Alcohol and older people. The European project vintage: Good Health Into Older Age. *Ann Ist Super Sanita, 48*(48), 221–231.

Garforth, C., Ozor, N., Usher, R., & Bell, A. (2014). *Global Development Network Independent Evaluation. Final Report*. New Delhi: Global Development Network.

Hanley, T., Gould, C., Harle, J., & Nelson, K. (2012). *International Network for the Availability of Scientific Publications. Programme for the Enhancement of Research Information. Phase II. External Evaluation 2008-12. Final Report.* Oxford: International Network for the Availability of Scientific Publications.

Hawkes, S., B., K. A., Jadeja, N., Jimenez, M., Buse, K., Anwar, I., . . . Whitworth, J. (2016). Strengthening capacity to apply health research evidence in policy making: experience from four countries. *Health Policy Plan, 31*(2), 161-170. doi:10.1093/heapol/czv032

Johnson, N. A., & Lavis, J. N. (2010). *Outcomes Evaluation. Procedures Manual for for Evaluating Knowledge-Translation Platforms in Low- and Middle- Income Countries.* (M. U. P. i. P. Decision-Making Ed.). Hamilton, Canada: McMaster University Program in Policy Decision-Making.

Kwan, P., Johnston, J., Fung, A. Y., Chong, D. S., Collins, R. A., & Lo, S. V. (2007). A systematic evaluation of payback of publicly funded health and health services research in Hong Kong. *BMC Health Serv Res, 7*, 121. doi:10.1186/1472-6963-7-121

Langlois, E. V., Becerril Montekio, V., Young, T., Song, K., Alcalde-Rabanal, J., & Tran, N. (2016). Enhancing evidence informed policymaking in complex health systems: lessons from multi-site collaborative approaches. *Health Res Policy Syst, 14*(20). doi:10.1186/s12961-016-0089-0

Mahmood, S., Hort, K., Ahmed, S., Salam, M., & Cravioto, A. (2011). Strategies for capacity building for health research in Bangladesh: Role of core funding and a common monitoring and evaluation framework. *Health Res Policy Syst, 9*(31).

Makkar, S. R., Turner, T., Williamson, A., Louviere, J., Redman, S., Haynes, A., . . . Brennan, S. (2016). The development of ORACLe: a measure of an organisation's capacity to engage in evidence-informed health policy. *Health Res Policy Syst, 14*, 4. doi:10.1186/s12961-015-0069-9

Mavoa, H., Waqa, G., Moodie, M., Kremer, P., McCabe, M., Snowdon, W., & Swinburn, B. (2012). Knowledge exchange in the Pacific: The TROPIC (Translational Research into Obesity Prevention Policies for Communities) project. *BMC Public Health, 12*, 552. doi:10.1186/1471-2458-12-552

Murnaghan, D., Morrison, W., Griffith, E. J., Bell, B. L., Duffley, L. A., McGarry, K., & Manske, S. (2013). Knowledge exchange systems for youth health and chronic disease prevention: a tri-provincial case study. *Chronic Diseases and Injuries in Canada, 33*(4), 257-266.

Neves, J., Lavis, J. N., Panisset, U., & Klint, M. H. (2014). Evaluation of the international forum on evidence informed health policymaking: Addis Ababa, Ethiopia – 27 to 31 August 2012. *Health Res Policy Syst, 12*(14).

Ongolo-Zogo, P., Lavis, J. N., Tomson, G., & Sewankambo, N. K. (2014). Initiatives supporting evidence informed health system policymaking in Cameroon and Uganda: a comparative historical case study. *BMC Health Services Research, 14*(612).

Ottoson, J. M., Green, L. W., Beery, W. L., Senter, S. K., Cahill, C. L., Pearson, D. C., . . . Leviton, L. (2009). Policy-contribution assessment and field-building analysis of the Robert Wood Johnson Foundation's Active Living Research Program. *Am J Prev Med, 36*(2 Suppl), S34-43. doi:10.1016/j.amepre.2008.10.010

Panel on Return on Investment in Health Research. (2009). *Making an Impact. A Preferred Framework and Indicators to Measure Returns on Investment in Health Research.* (C. A. o. H. Sciences Ed.). Ottawa, ON: Canadian Academy of Health Sciences.

Peirson, L., Catallo, C., & Chera, S. (2013). The Registry of Knowledge Translation Methods and Tools: a resource to support evidence-informed public health. *Int J Public Health, 58*(4), 493-500. doi:10.1007/s00038-013-0448-3

R. Makkar, S., Brennan, S., Turner, T., Williamson, A., Redman, S., & Green, S. (2016). The development of SAGE: A tool to evaluate how policymakers’ engage with and use research in health policymaking. *Research Evaluation, 25*(3), 315-328. doi:10.1093/reseval/rvv044

Sallis, J. F., Linton, L. S., Kraft, M. K., Cutter, C. L., Kerr, J., Weitzel, J., . . . Pratt, M. (2009). The Active Living Research program: six years of grantmaking. *Am J Prev Med, 36*(2 Suppl), S10-21. doi:10.1016/j.amepre.2008.10.007

Sources4Network. (2016). Network Maturity Matrix. In. London: NHS England [website]. https://www.source4networks.org.uk/images/site/files/Maturity_Model_Matrix_v2_071216-FINAL.pdf.

The CIPHER Investigators. (2014). Supporting Policy In health with Research: an Intervention Trial (SPIRIT)-protocol for a stepped wedge trial. *BMJ Open, 4*(7), e005293. doi:10.1136/bmjopen-2014-005293

The Networks of Centres of Excellence Secretariat. (2008). *Joint Results-based Management and Accountability Framework and Risk-Based Audit Framework for the Class Grant Networks of Centres of Excellence Program*. Ottawa: The Networks of Centres of Excellence Secretariat.

Uneke, C. J., Ezeoha, A. E., Ndukwe, C. D., Oyibo, P. G., & Onwe, F. (2011). Enhancing health policymakers’ capacity to use information and communication technology in Nigeria. *Health Inform Dev Ctries, 5*(2), 228–246.

Uneke, C. J., Ezeoha, A. E., Uro-Chukwu, H., Ezeonu, C. T., Ogbu, O., Onwe, F., & Edoga, C. (2015a). Enhancing the Capacity of Policy-Makers to Develop Evidence-Informed Policy Brief on Infectious Diseases of Poverty in Nigeria. *Int J Health Policy Manag, 4*(9), 599-610. doi:10.15171/ijhpm.2015.100

Uneke, C. J., Ezeoha, A. E., Uro-Chukwu, H., Ezeonu, C. T., Ogbu, O., Onwe, F., & Edoga, C. (2015b). Improving Nigerian health policymakers' capacity to access and utilize policy relevant evidence: outcome of information and communication technology training workshop. *Pan Afr Med J, 21*, 212. doi:10.11604/pamj.2015.21.212.6375

Vogel, I., & Punton, M. (2016). *Building Capacity to Use Ressearch Evidence (BCURE) Evaluation: Stage 1 Synthesis Report*. Brighton: Itad.

Vogel, I., & Punton, M. (2017). *Building Capacity to Use Research Evaluation (BCURE) realist evaluation: Stage 2 Synthesis Report*: ITAD.

Waqa, G., Mavoa, H., Snowdon, W., Moodie, M., Nadakuitavuki, R., Mc Cabe, M., & Swinburn, B. (2013). Participants’ perceptions of a knowledge-brokering strategy to facilitate evidence-informed policy-making in Fiji. *BMC Public Health, 13*(725).

Waqa, G., Mavoa, H., Snowdon, W., Moodie, M., Schultz, J., McCabe, M., . . . Swinburn, B. (2013). Knowledge brokering between researchers and policymakers in Fiji to develop policies to reduce obesity: A process evaluation. *Implement Sci, 8*(74).

Yazdizadeh, B., Majdzadeh, R., Alami, A., & Amrolalaei, S. (2014). How can we establish more successful knowledge networks in developing countries? Lessons learnt from knowledge networks in Iran. *Heal Res Policy Syst, 12*(63).
